# Cellular correlates of gray matter volume changes in magnetic resonance morphometry identified by two-photon microscopy

**DOI:** 10.1038/s41598-021-83491-8

**Published:** 2021-02-19

**Authors:** Livia Asan, Claudia Falfán-Melgoza, Carlo A. Beretta, Markus Sack, Lei Zheng, Wolfgang Weber-Fahr, Thomas Kuner, Johannes Knabbe

**Affiliations:** 1 Department of Functional Neuroanatomy, Institute for Anatomy and Cell Biology, Heidelberg University, Im Neuenheimer Feld 307, 69120 Heidelberg, Germany; 2 Translational Imaging Research Group, Central Institute of Mental Health, Medical Faculty of Mannheim, Heidelberg University, J5, 68159 Mannheim, Germany; 3 CellNetworks Math-Clinic, Heidelberg University, Bioquant BQ001, Im Neuenheimer Feld 267, 69120 Heidelberg, Germany; 4 Present Address: Center for Psychosocial Medicine, Department of General Psychiatry, Heidelberg University Hospital, Voßstraße 4, 69120 Heidelberg, Germany

**Keywords:** Cellular neuroscience, Diseases of the nervous system, Neural ageing, Brain, Translational research, Preclinical research, Neuroscience

## Abstract

Magnetic resonance imaging (MRI) of the brain combined with voxel-based morphometry (VBM) revealed changes in gray matter volume (GMV) in various disorders. However, the cellular basis of GMV changes has remained largely unclear. We correlated changes in GMV with cellular metrics by imaging mice with MRI and two-photon in vivo microscopy at three time points within 12 weeks, taking advantage of age-dependent changes in brain structure. Imaging fluorescent cell nuclei allowed inferences on (i) physical tissue volume as determined from reference spaces outlined by nuclei, (ii) cell density, (iii) the extent of cell clustering, and (iv) the volume of cell nuclei. Our data indicate that physical tissue volume alterations only account for 13.0% of the variance in GMV change. However, when including comprehensive measurements of nucleus volume and cell density, 35.6% of the GMV variance could be explained, highlighting the influence of distinct cellular mechanisms on VBM results.

## Introduction

Magnetic resonance imaging (MRI) has tremendously advanced our understanding of brain structure and function in health and disease. Brain morphology and tissue composition can be examined using a variety of structural scanning protocols^1^. A range of automated image analysis tools providing unbiased results have been developed in the past decades to quantify structural changes. One of the most widely used computational approaches is voxel-based morphometry (VBM)^2,3^. VBM provides an automated quantitative analysis of the distribution of gray and white matter to detect differences in brain tissue concentration for each voxel (e.g. gray matter density, GMD). To include voxel-wise volume changes, the GMD is then modulated by multiplication with the Jacobian Determinant (JD). The JD is derived from the non-linear deformation field needed to transform each subject brain to a given template brain^4^. The modulated GMD is then multiplied with the voxel volumes and is interpreted as gray matter volume (GMV).

**Figure 1:**
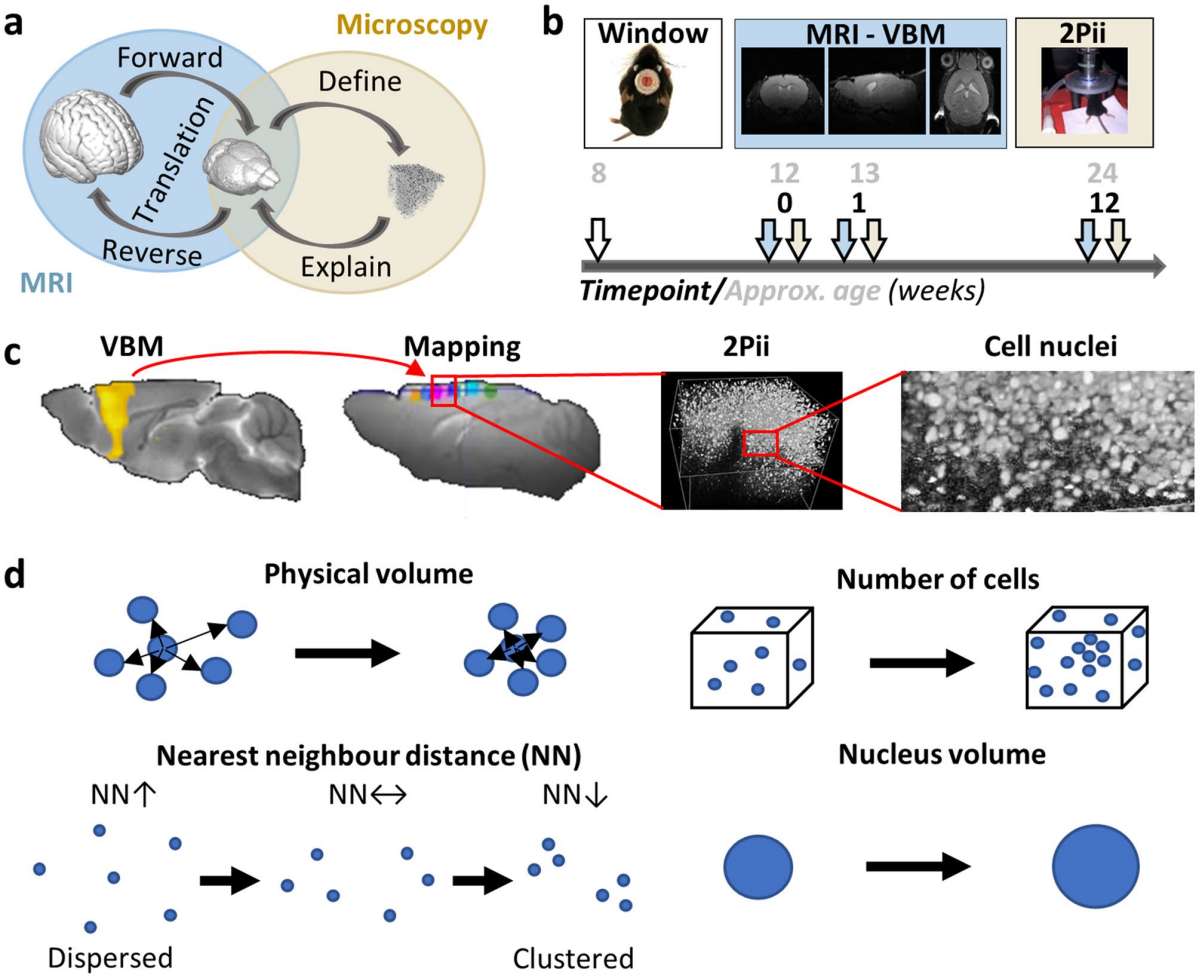
Investigating cerebrocortical structure across species, size and time. (**a**) Using MRI in human and rodent studies, findings between species with the same imaging modality can be directly translated. Explorative whole brain studies identify key regions which can be further investigated by microscopy studies in mice, revealing specific cellular mechanisms for large-scale structural observations. (**b**) MRI and 2Pii allow for repetitive imaging in vivo and therefore capture dynamics of brain structure in individual animals. The imaging intervals were precisely defined in each mouse (time points given in black), while the corresponding age of the mice may differ ± 1 week due to the staggered experimental design. (**c**) VBM results shown on brain template, mapping of MRI volumes to 2Pii volumes, 3D 2Pii stack and zoomed in view showing the distribution of cell nuclei. (**d**) Parameters determined from imaging cell nuclei in 3D space. Further details see main text.

VBM has been applied to examine physiological aging^5^ as well as a vast spectrum of diseases such as depression^6,7^, panic disorder^8^, posttraumatic stress disorder^9^, chronic pain^10^ and Alzheimer’s disease^11^. Translational studies have become possible by extending VBM to animal studies, leading to a better understanding of pathology-related changes in brain structure^12–14^. Despite the widespread application of VBM, the physical basis of the terms “tissue concentration” or “volume”, commonly used by VBM studies to describe structural changes, remained poorly defined and mostly serve as semantic wild cards. However, a mechanistic understanding of the physical and cellular basis of GMV changes is pertinent (Fig. 1a)^15–17^.

Potential mechanisms underlying volumetric changes detected with VBM have been proposed to include altered glial proliferation or neurogenesis, changes in neuronal or glial size, angiogenesis and endothelial cell proliferation, shifts in dendritic spine size and density, remodelling of axonal processes or alterations of the extracellular space (e.g. by changes in blood-perfusion and ultrafiltration, edema, glymphatic system)^18–20^. To address the lack of mechanistic understanding, few studies have been conducted in animals to directly compare the MRI-detectable volume changes with detailed ex vivo histological analyses^18,19,21^. These studies mostly focused on changes in spine density as well as markers for newborn neurons and astrocytes in the mouse hippocampus.

Studies comparing VBM changes with ex vivo cellular assessments are typically limited to single time points and lack the ability to follow changes within individual subjects. To overcome these limitations, we designed a longitudinal translational neuroimaging approach that combines, in the same mice, structural MRI and two-photon in vivo imaging (2Pii), a microscopy technique well suited to image cortical volumes through implanted cranial windows (Fig. 1b^22^). As there are numerous possible cellular mechanisms for GMV change, we aimed to obtain a general, comprehensive readout to validate physical tissue volume changes on a microscopic level as well as gain additional information about cellular architecture (Fig. 1c). For this purpose, we chose to visualize cell nuclei in the cortex by imaging brains of mice expressing green fluorescent protein in all cell nuclei. We used repetitive longitudinal imaging of cell nuclei to determine (Fig. 1d): (i) a defined physical three-dimensional space (i.e. a tissue volume given in µm^3^) to quantify tissue shrinkage or expansion; (ii) nucleus count and therefore cell density; (iii) the distances to the neighboring nuclei to measure the degree of cell clustering; and (iv) the mean volume of the nuclei as a putative indicator for cell type composition changes or changes in transcriptional activity. These parameters were individually correlated to the changes in VBM-derived measures that occurred during 12 weeks.

## Results

**Figure 2:**
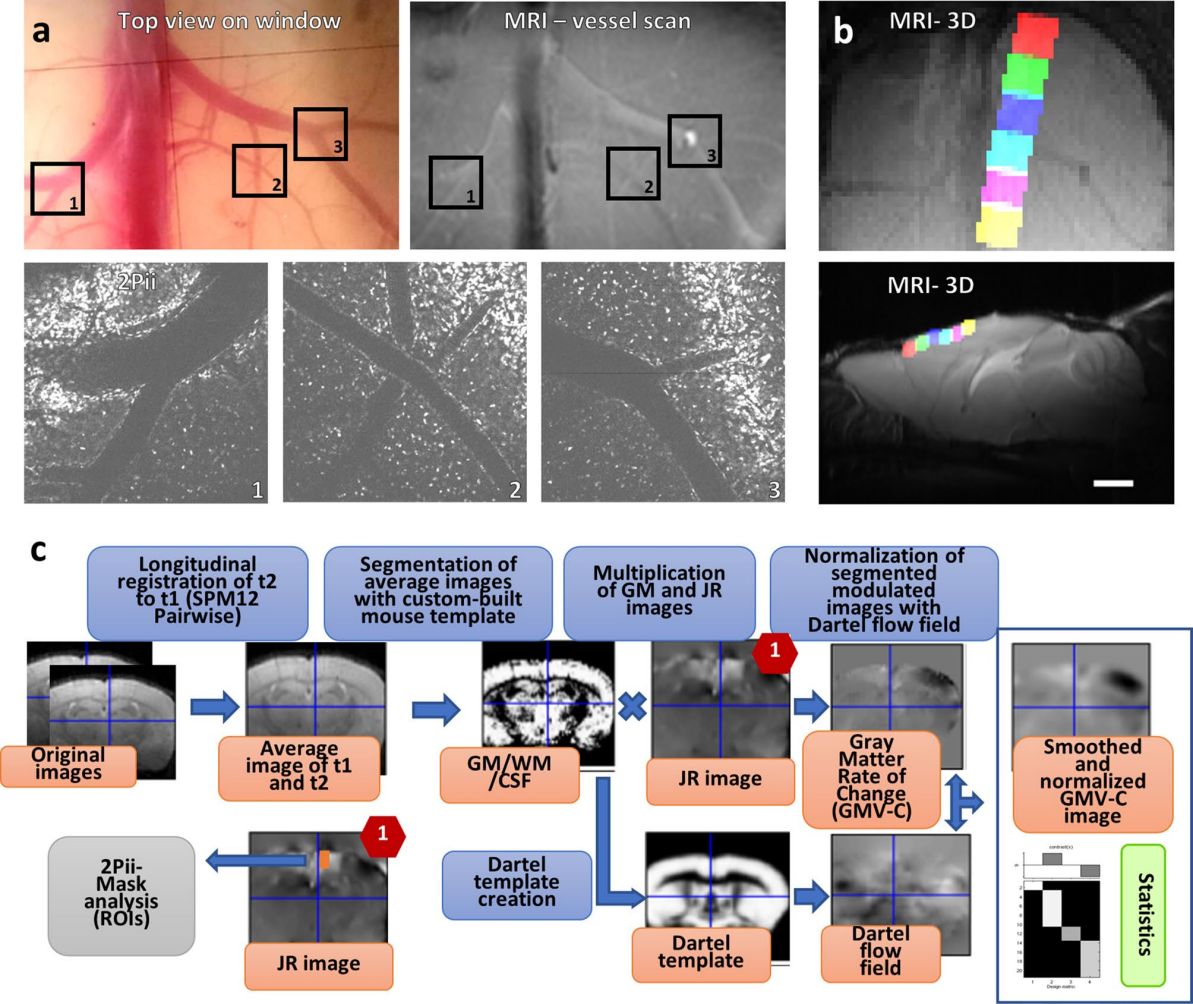
Cross-modal affine image registration using vessel branchings as fiducial points and VBM analysis workflow. (**a**) Vessel branchings are visible in MRI as well as 2Pii without any additional contrast agent. Upper left panel shows view on exposed brain surface after window surgery, regions 1–3 indicate the positions of vessel branches in MRI and 2Pii. (**b**) Six partially overlapping 2Pii-stack-masks of one animal registered on the corresponding MR image. Average intensity projections; up: horizontal view, bottom: sagittal view. Scale bar = 2 mm. (**c**) Longitudinal registration and processing of MRI 3D images for VBM. Longitudinal nonlinear registration was performed for each subject between timepoints (t1 = baseline, t2 = 1 week or 12 weeks), which yielded resulting average and Jacobian Rate (JR) images. The average images were pre-segmented in three tissue probability maps [gray matter (GM), white matter (WM), cerebrospinal fluid (CSF)] and multiplied by the JR-images. From these, a Dartel prior knowledge template was created and the resulting flow fields were used to normalize the JR-modulated tissue maps into Paxinos space. They were finally smoothed and statistically analysed in SPM second-level models.

The longitudinal imaging scheme started 4 weeks after implantation of the cranial windows, when mice were approximately 12 weeks old. A baseline MRI dataset and a 3D 2Pii dataset consisting of six imaging positions within the cortex per animal were acquired, followed by a second dataset after 1 week and a third dataset after 12 weeks (Fig. 1b). Cross-modal registration of image volumes was achieved by mapping the blood vessel patterns, providing a basis to define masks matching the 2Pii positions within the field of view of MRI for quantitative comparisons of the two modalities (Fig. 2a,b; “Methods”). The longitudinal VBM workflow was designed to find changes in GMV at the two later timepoints compared to the baseline measurement (Fig. 2c; “Methods”). The natural brain curvature was preserved by using curved glass cover slips as cranial windows (Supplementary Figure 1).

### VBM analysis

**Figure 3:**
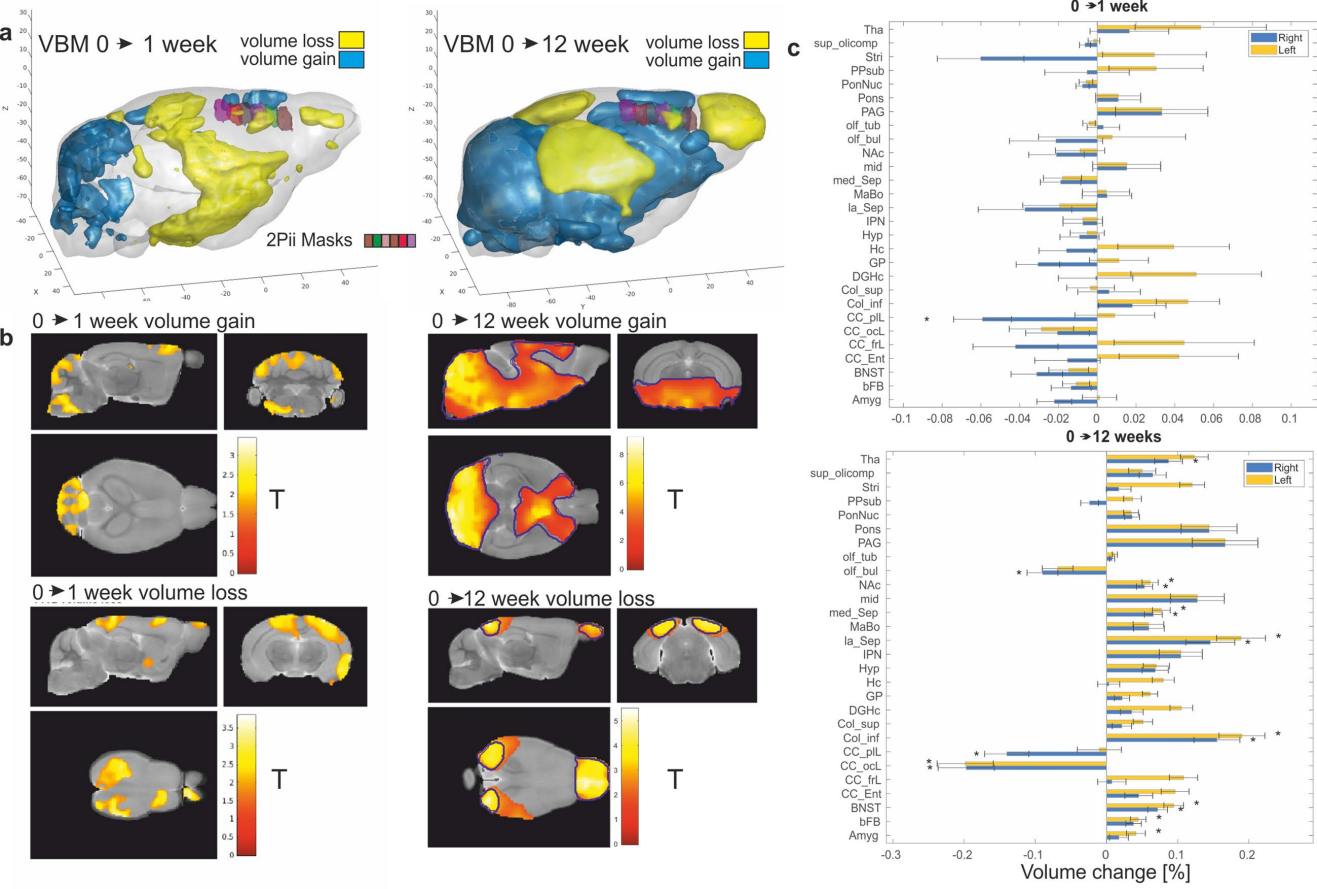
Longitudinal assessment of brain volume changes with VBM. (**a**) Three-dimensional representation with positive (blue) and negative (yellow) volume changes observed at 1 week and 12 weeks after baseline (p < 0.05 uncorrected for display purposes). Imaging volumes addressed with 2Pii are labeled with distinct colours (see legend in left panel). Tick spacing is 20 mm on all axes. (**b**) Statistical maps depicting areas exhibiting significant volume growth or loss (p < 0.05 uncorrected for display purposes, significant at p < 0.05 cluster correction, the blue contour line depicts areas which are significant after TFCE FDR-correction). Color bar indicates t-value. (**c**) Volume changes of 28 anatomical regions over time assessed by longitudinal VBM between the baseline measurement vs. 1 and 12 weeks in % from baseline. Regions with significant changes from baseline (p < 0.05) are marked (*). Error bars indicate SEM. Abbreviations: Amygdala (Amyg), basal forebrain (bFB), bed nucleus of stria terminalis (BNST), entorhinal cortex (CC_Ent), frontal lobe (CC_frL), occipital lobe 130 164 (CC_ocL), parieto-temporallobe (CC_ptL), colliculus: inferior (Col_inf), colliculus superior (Col_sup), dentate gyrus of hippocampus (DGHC), globus pallidus (GP), hippocampus (HC), hypothalamus (Hyp), interpeduncular nucleus (IPN), lateral septum (la_Sep), mammillary bodies (MaBo), medial septum (med_Sep), midbrain (mid), nucleus accumbens (NAc), olfactory bulbs (olf_bul), olfactory tubercle (olf_tub), periaqueductal gray (PAG), pons (Pons), pontine nucleus (PonNuc), pre-para subiculum (PPSub), striatum (Stri), superior olivary complex (sup_olicomp) and thalamus (Tha).

VBM results are visualized in Fig. 3. Regions within the cerebellum and midbrain increased in GMV during the first week, while areas that primarily lie in the parietal lobe and parts of the frontal lobe showed GMV reduction. After 12 weeks, this pattern became more pronounced with widespread GMV increases in the cerebellum and thalamic areas. In contrast, especially the olfactory bulb, occipital and parietal lobes decreased in GMV with most pronounced changes occurring in visual areas. The frontal and prefrontal areas initially show a trend of lateralization, with GMV loss on the right and a slight gain on the left side at 1 week. This divergence was largely compensated during the following weeks, showing a trend for volume gains in these regions at 12 weeks (Fig. 3a,b). The main effect after 12 weeks was identified as a volume gain in the cerebellum and visual areas as well as a distinct volume loss in the olfactory bulb. While the voxel-based GMV changes during the first week were not significant after correction for multiple comparisons, all shown clusters with changes after 12 weeks were significant after false discovery rate (FDR) corrected threshold free cluster enhancement (TFCE) with 5000 permutations. The quantified volume changes are shown in Fig. 3c for 28 atlas regions^23^. The general pattern of changes described here was similar to a previous study probing age-dependent GMV changes, with the difference that the mean alteration of volume was more shifted to GMV loss which may be due to the different age range investigated in this study^14^. Our regions of interest in this study, the imaging sites of 2Pii covering the cingulate cortex, were located on the right brain hemisphere and are superimposed in Fig. 3a as well as in Supplementary Figure 6. A tendency of GMV decrease was evident at 1 week after baseline, but 11 weeks later, this effect seems to have partly reversed, with areas within the 2Pii masks that vary between increased and decreased GMV.These voxel-wise results are reflected in the statistics over the averaged GMV volumes for each 2Pii mask depicted below (Fig. 6c). While VBM shows a significant GMV decrease within the first week, the results are less pronounced at 12 weeks, owing to an increase in variance over the individual animals. Any voxel-wise comparison between the two imaging modalities needs to consider that surrounding tissue areas affect the MRI measurements derived from of each voxel, owing to the process of coregistration and smoothing during VBM (see “Methods” and “Discussion”).

In summary, we found changes in GMV over time in distinct areas, including superficial regions of the neocortex that are accessible to 2Pii, thereby providing a basis for the correlative approach described here.

### Imaging cell nuclei to derive structural parameters for cortical volumes

**Figure 4:**
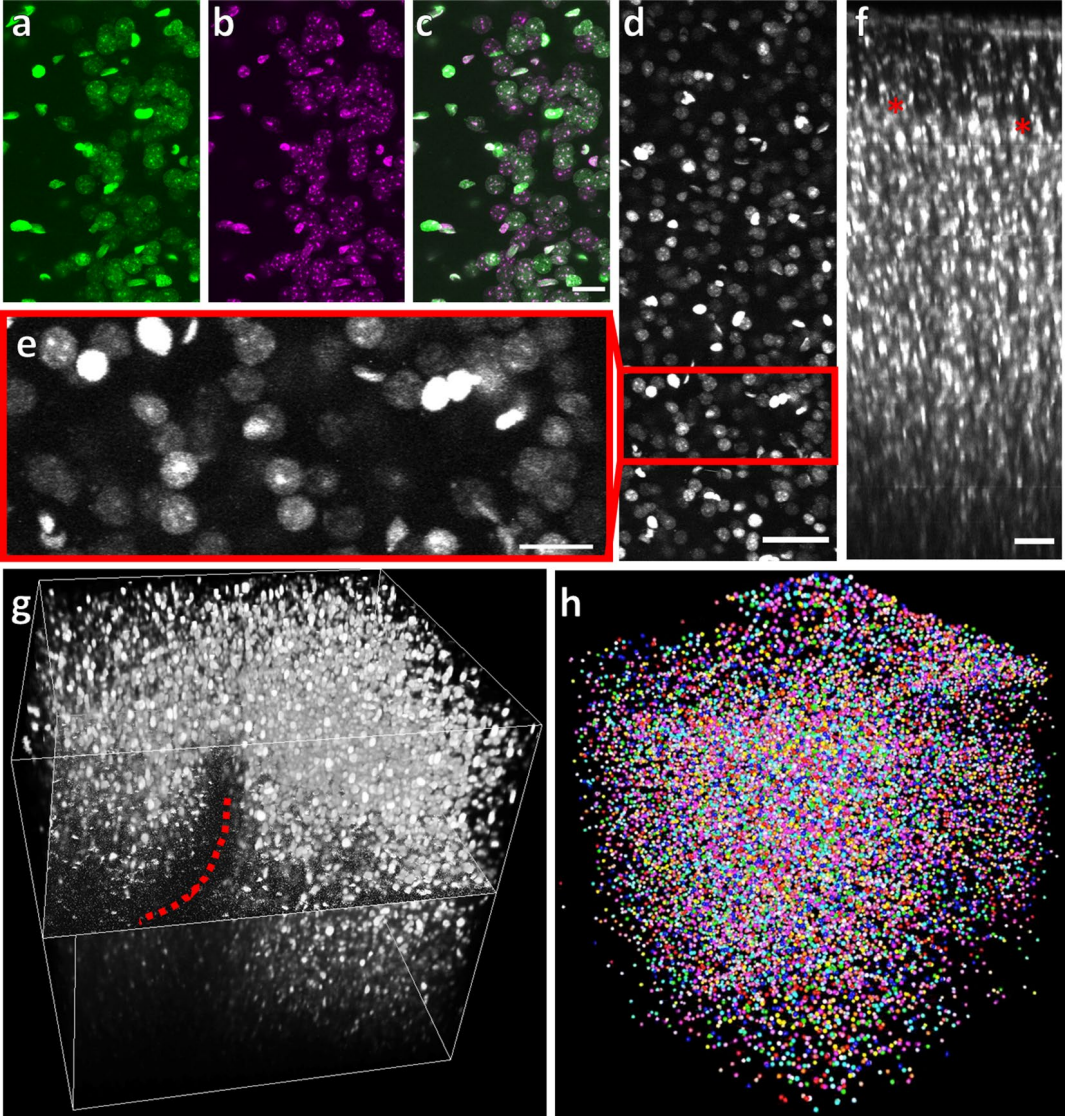
Histone-GFP labeled cell nuclei. (**a**–**c**) Confocal Microscopy of fixed brain slices of Histone-GFP mice shows that all DAPI-stained nuclei (**a**) are also labeled with Histone-GFP (**b**) as seen in the overlap (**c**, green = Histone-GFP, magenta = DAPI). Scale bar = 10 µm. (**d**) Representative example image consisting of a maximum intensity projection of 10 consecutive image frames covering 20 µm of tissue recorded at a depth of 200 µm in Histone-GFP positive mice using 2Pii. Scale bar = 100 µm. (**e**) Close up of D. Scale bar = 20 µm. (**f**) Average intensity projection of a stack cropped in ZX. The surface of the brain is located at the top of the image. Red stars delineate the lower border of cortical layer 1 as visible in change of cell density. Scale bar = 50 µm. (**g**) A representative rendering of a 700 µm × 700 µm × 700 µm stack recorded by in vivo 2Pii in a Histone-GFP mouse. One orthogonal slice is displayed in the middle of the stack. The red stippled line denotes the ‘shadow’ cast by a blood vessel which strongly attenuates the laser beam. (**h**) Detected nuclei within a volume of 700 µm × 700 µm × 700 µm. Each sphere corresponds to the centroid position of a nucleus and is randomly coloured.

To identify cell nuclei in vivo, we used genetically modified mice which ubiquitously express an EGFP-tagged Histone-H2B protein (‘Histone-GFP’, see “Methods”). The extent of labeling was tested by staining fixed brain sections with DAPI, an established reagent to stain DNA. Confocal imaging of Histone-GFP and DAPI revealed co-labeling of all nuclei, suggesting that in Histone-GFP mice all nuclei were labeled (Fig. 4a–c).

A representative image recorded with 2Pii through the cranial window at a depth of 200 µm shows densely packed nuclei (Fig. 4d). The magnified view highlights that nuclei were often sub-structured and of different size and shape (Fig. 4e). A typical image stack reveals layers differing in the density of nuclei, consistent with the layered structure of the neocortex (Fig. 4f). The most superficial densely packed nuclei are likely to be part of the pial membrane and astrocytes contributing to the glial limiting membrane. The border between the molecular layer and layer 2/3 could be readily identified (asterisks in Fig. 4f). A 3D projection of a complete image stack covering 700 µm × 700 µm × 700 µm shows the full extent of the volume acquired by one stack in 2Pii (Fig. 4g).

In summary, these results show that 2Pii of nuclei in neocortical areas corresponding to defined MRI volumes is feasible and delivers image data qualitatively suitable for longitudinal analyses as further outlined below. For instance, these image data were processed by an automated pipeline (see “Methods”) and resulted in a 3D matrix of centroid positions each reflecting the location of a nucleus (Fig. 4h, Supplementary Figure 2).

### Physical volume can be inferred from convex hull volumes spanned by manually identified marker nuclei

**Figure 5:**
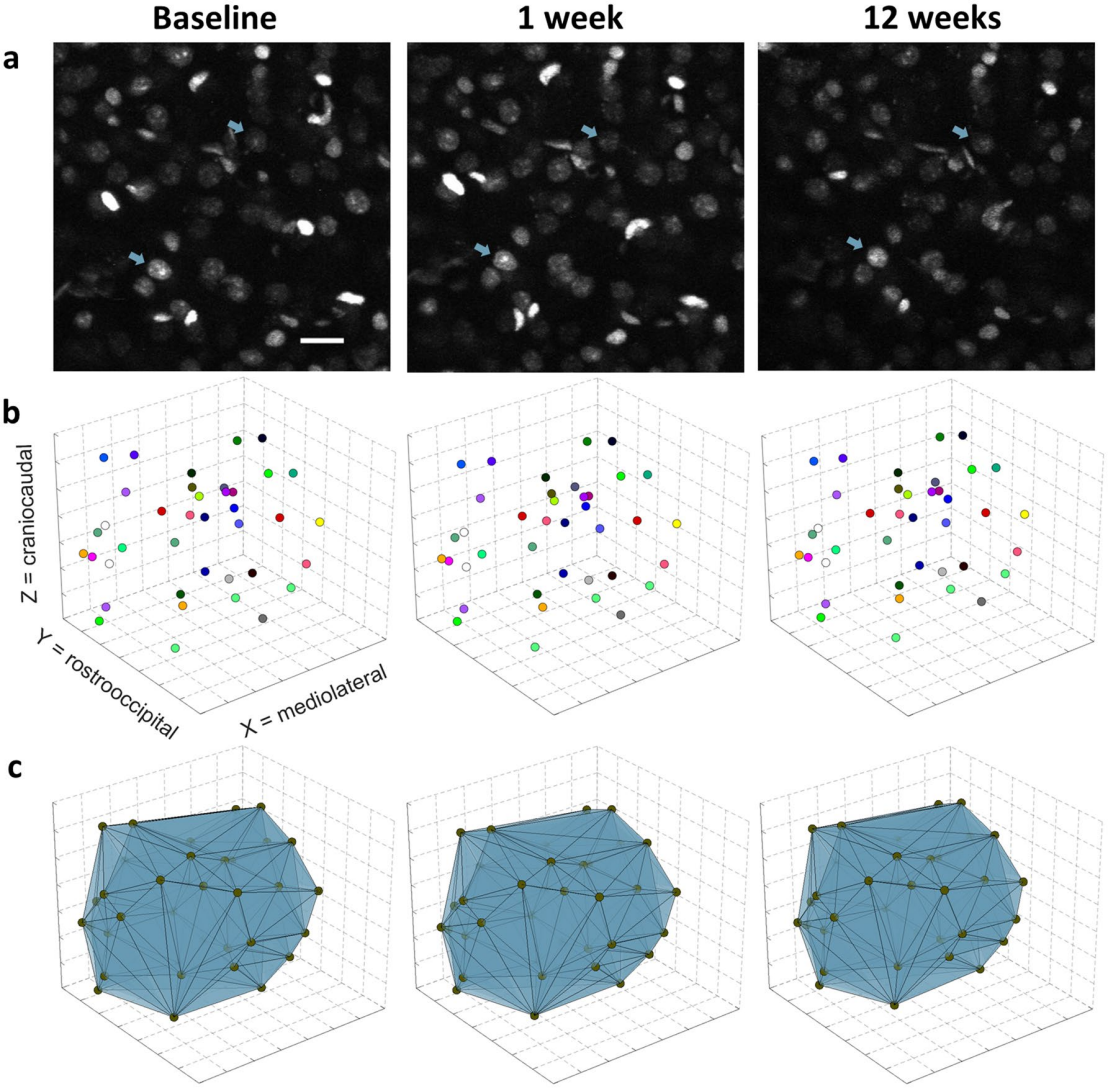
Determination of physical tissue volume from a convex hull spanned between nuclei reidentified over time. (**a**) Stable patterns of nuclei were reidentified over all timepoints and nucleus centre coordinates were sampled as fiducial markers throughout all stack dimensions. Two examples of reidentified nuclei are indicated by blue arrows. Maximum intensity Z-projection of 10 optical sections covering 20 µm. Scale bar = 20 µm. (**b**) Fiducial marker coordinates plotted inside single stack volume. Dot colours specify fiducials that could be reidentified reliably at all three time points. Grid lines spaced in 100 µm. (**c**) Three-dimensional Delaunay triangulation from fiducials create matching tetrahedra. All tetrahedra create a convex hull (entire volume in blue) whose subtle volume changes indicate shrinkage or expansion of the tissue within.

To identify changes of physical cortical volume from the positions of cell nuclei within a 2Pii stack, we developed a method considering several important confounding factors (see “Methods”). On the timescale of the imaging intervals, the position of a detected nucleus could not be assumed to be stable, because cell death, proliferation and migration may change the composition and geometrical arrangement of nuclei without affecting the local tissue volume. To circumvent these problems, we manually searched for nuclei that could (a) be unequivocally found and reidentified at all three timepoints and (b) serve as markers for stationary cells. Through inspection of 2Pii image data, we indeed found that many nuclei stayed in distinctly recognizable, stable local patterns with other neighboring nuclei over many months (Fig. 5a). As it seems unlikely that this whole pattern of cells actively migrates together, we assumed that these reidentifiable nuclei represent stationary cells which keep their positions stable in the tissue. We sampled the xyz coordinates of a set of such nuclei distributed in all stack dimensions at all experimental time points and constructed the volume spanned between them, called the convex hull (Fig. 5b,c). Following our rationale, volume shifts in the convex hull can be interpreted as a shrinkage or expansion of the tissue between the reidentified, stable nuclei (see “Methods”). A concerted migration of a subset of nuclei into different directions, which would confound our volume readout, seems highly unlikely. We sampled convex hulls in three of the six imaged positions in every mouse. The volume of the convex hulls was in the range of 0.03–0.12 mm^3^ and was defined by the arbitrary choice of identified nuclei rather than reflecting the total scanned image volume.

### Correlations of GMV with physical volume changes

**Figure 6:**
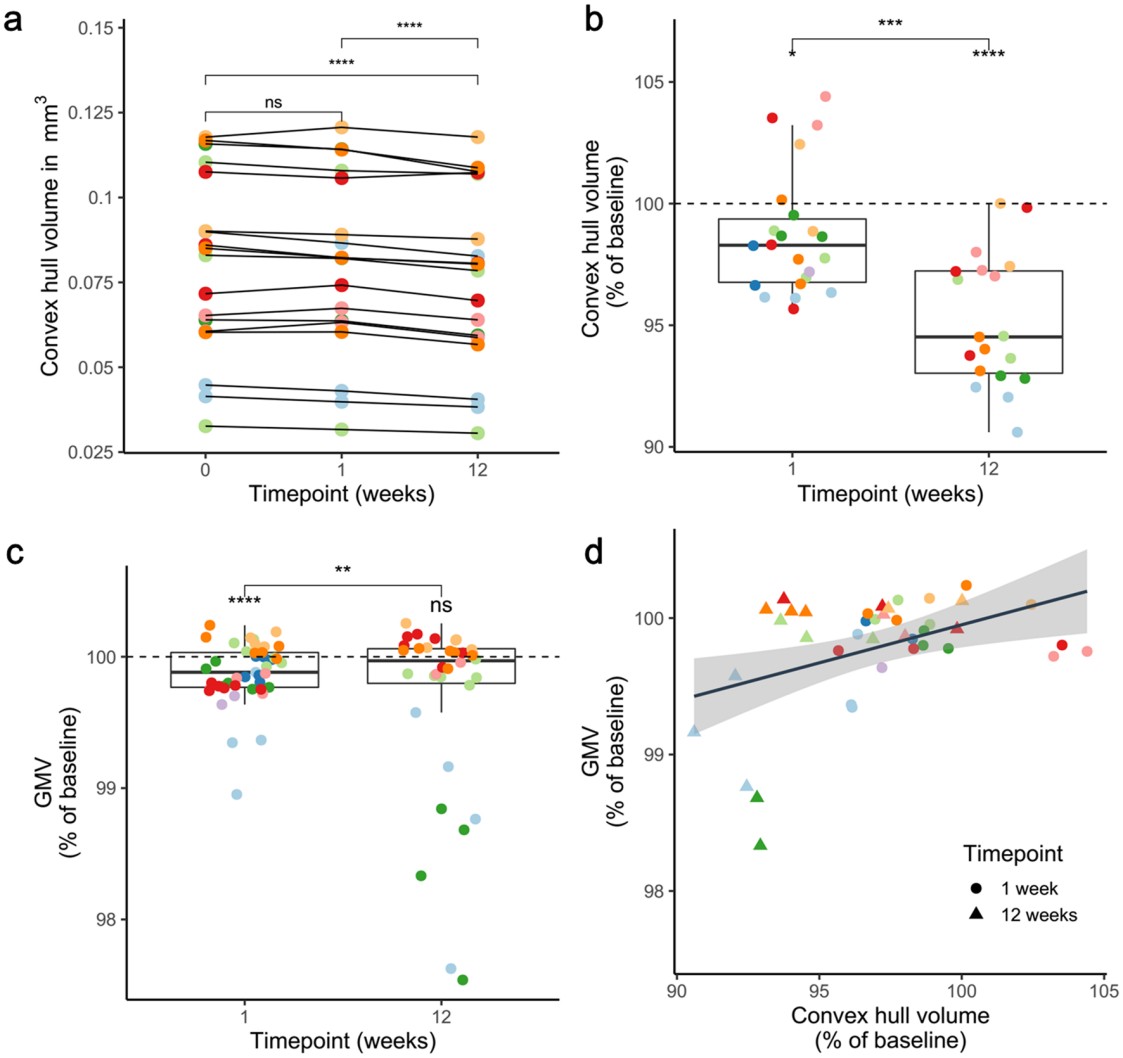
Correlation of tissue volume with GMV. Colours of plotted datapoints denote individual mice. (**a**) Volumes of convex hulls in 2Pii stacks significantly shrink over time. Display of all measured absolute volumes. Repeated measure one-way ANOVA with complete datasets (all timepoints available) of n = 18 positions of 7 mice, F = 29.12, DF = 2, p =  < 0.0001. Post-hoc Tukey test for multiple comparisons with adjusted p values: 0 vs. 1 week: p = 0.260; 0 vs. 12 weeks: p < 0.0001; 1 vs. 12 weeks: p < 0.0001. (**b**) Relative change in 2Pii convex hull volumes compared to baseline (= theoretical mean of 100%) at 1 week and 12 weeks. In addition to Fig. 6A, also datapoints with missing values for the 12 weeks timepoint are included. At 1 week: n = 22 positions of 9 mice, mean ± SD = 98.74 ± 2.55%; Wilxocon signed rank test gives p = 0.046. At 12 weeks n = 19 positions of 7 mice, mean ± SD = 95.16 ± 2.71%, Wilxocon signed rank test gives p < 0.0001; linear mixed effect model for difference between 1 and 12 weeks: p < 0.0001. (**c**) Changes of GMV in MRI masks of 2Pii stacks. Wilxocon signed rank test at 1 week gives mean ± SD = 99.87 ± 0.24%, p < 0.001, n = 45 positions of 9 mice; at 12 weeks: mean ± SD = 99.67% ± 0.71%, p = 0.075, n = 35 positions of 7 mice. Linear mixed effect model for difference 1 week and 12 weeks: p = 0.008. (**d**) Microscopic volume changes correlate with GMV in MRI masks. Linear mixed effect model with p = 0.014, Estimate = 0.046%, R^2^ = 0.130, n = 41 (22 positions of 9 mice at 1 week, 18 positions of 7 mice at 12 weeks).

The convex hull volumes revealed a successive decrease in tissue volume from baseline to 1 and 12 weeks (Fig. 6a,b). At week 12, the volume decreased to 95% of baseline (Fig. 6b), suggesting a shrinkage of the tissue by 5%. When analyzing GMV changes in regions of interest which are defined by MRI masks corresponding to the location of 2Pii stacks (see “Methods” for details), we found a significantly decreased GMV relative to baseline at week 1 and between week 1 and 12, but not at week 12 when compared to baseline (Fig. 6c). In a linear mixed-effect model, enlargement of the convex hull volume significantly correlated with GMV gain, with an R^2^ of 0.130, indicating that the variance in GMV explained by this microscopic volume readout amounted to 13% (Fig. 6d).

### Correlations of GMV with the number of cells

We next tested if changes in the number of nuclei within a 2Pii stack correlated with alterations in the respective GMV. The number of nuclei and all subsequent readouts were derived from our automated image analysis for 3D nucleus detection and segmentation (see “Methods”, Supplementary Figure 2 and Video 1), which allowed us to investigate more datasets than we did for manual determination of physical tissue volume.

**Figure 7:**
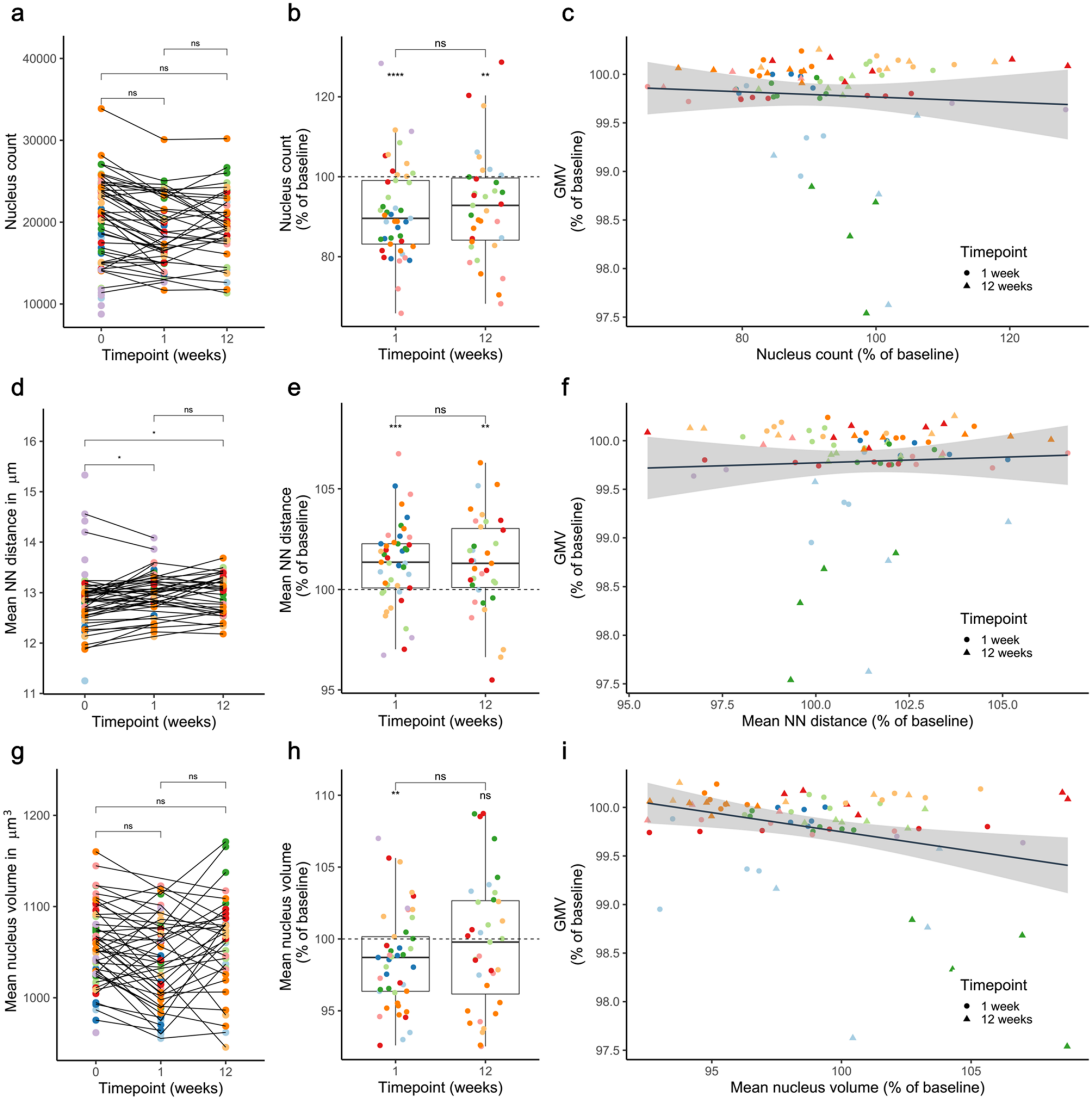
Correlations of GMV with cell number, nearest neighbour distance and mean nucleus volume. (**a**,**d,g**) n = 60 positions from 10 mice in week 0, 45 positions from 9 mice in week 1, 35 positions from 7 mice in week 12. (**b**,**c**,**e**,**f**,**h**,**i**) At 1 week: n = 45 positions from 9 mice; at 12 weeks: n = 35 positions of 7 mice. (**a**) Number of cells within the whole 2Pii stack as counted by automated detection. Mean ± SD over all 2Pii stacks is given in thousands: baseline = 19.50 ± 5.46; 1 week = 18.70 ± 4.31, 12 weeks = 19.77 ± 4.31. Linear mixed effect model with pairwise contrasting and correction for multiple comparison: week 1 vs. baseline: p = 0.12, week 12 vs. baseline: p = 0.55, week 12 vs. week 1: p = 0.73. (**b**) Relative change in the number of cells compared to baseline. Mean ± SD over all 2Pii stacks is given in % of baseline: 1 week = 91.26 ± 11.70; One sample *t* test gives p < 0.001, DF = 44, t = -5.009. 12 weeks = 92.72 ± 13.30; one-sample *t* test gives t = -3.24, DF = 34, p = 0.003. Linear mixed effect model for difference between 1 and 12 weeks: p = 0.430. (**c**) Correlation of GMV change and change of cell count. Linear mixed effect model: p = 0.303. (**d**) Mean nearest neighbour distance. Mean ± SD over all 2Pii stacks in µm: baseline = 12.89 ± 0.67; 1 week = 12.97 ± 0.42, 12 weeks = 12.96 ± 0.39. Linear mixed effect model with pairwise contrasting and correction for multiple comparison: week 1 vs. baseline: p = 0.030, week 12 vs. baseline: p = 0.025, week 12 vs. week 1: p = 0.950. (**e**) Relative change in nearest neighbour distances compared to baseline. Mean ± SD over all 2Pii stacks is given in % of baseline. 1 week: = 101.27 ± 2.05; one-sample *t* test gives t = 4.145, DF = 44, p < 0.001. 12 weeks = 101.27 ± 2.39; one-sample *t* test gives t = 3.156, DF = 34, p = 0.003. Linear mixed effect model for difference between 1 and 12 weeks: p = 0.871. (**f**) Correlation of GMV change with change of NN distance. Linear mixed effect model: p = 0.411. (**g**) Mean nuclear volume. Mean ± SD over all 2Pii stacks is given in µm^3^: baseline = 1054 ± 43; 1 week = 1039 ± 47; 12 weeks = 1060 ± 53. Linear mixed effect model with pairwise contrasting and correction for multiple comparison: week 1 vs. baseline: p = 0.167, week 12 vs. baseline: p = 0.962, week 12 vs. week 1: p = 0.155. (**h**) Relative change in mean nucleus volume compared to baseline. Mean ± SD over all 2Pii stacks is given in % of baseline. 1 week = 98.54 ± 3.34; one-sample *t* test gives t = -2.93, DF = 44, p = 0.005. 12 weeks = 99.67 ± 4.59; one-sample *t* test gives t = -0.43, DF = 34, p = 0.670. Linear mixed effect model for difference between 1 and 12 weeks: p = 0.080. (**i**) Correlation of GMV and mean nucleus volume. Linear mixed effect model: p = 0.002, Estimate = − 0.039%, R^2^ = 0.091.

The 3D nucleus detection algorithm revealed on average 19,504 ± 5457 nuclei per stack volume at baseline (n = 10 animals with 60 stack positions, Fig. 7a), with a pronounced variability between areas and mice. This variability is caused by the different extent and size of blood vessels contained in the imaging volumes (see Fig. 4g and “Discussion”). The total number of nuclei in a 2Pii stack did not significantly differ in the linear mixed model, only when stacks were analysed as independent datapoints and normalized to their baseline value the count was decreased at 1 and 12 weeks (Fig. 7b). The individual dynamics in cell number did not correlate with GMV changes (Fig. 7c).

### Correlations of GMV with nearest neighbor distances

The nearest neighbor (NN) distance reflects the distance between the centroid position of each nucleus to that of the nucleus nearest to it in Euclidean space. The mean NN distance of all NN distances in a stack was calculated. Changes in NN may be induced by different mechanisms acting in parallel: (i) uniform expansion or shrinkage of local tissue volume, with tissue expansion generating larger NN; (ii) gain or loss of cells in the immediate space surrounding a nucleus. For example, loss of the nearest neighbor would increase NN; (iii) a different distribution of nuclei, for example local clustering would decrease NN. The mean NN over all stacks was 12.89 µm at baseline, with an increase to 12.97 µm after 1 week. At week 12, NN was 12.96 µm, suggesting that NN remains unchanged after week 1. Hence, a small increase in NN distances could be detected (Fig. 7d,e). Correlating NN to GMV did not reveal a significant relationship (Fig. 7f).

### Correlations of GMV with nucleus volume

**Figure 8:**
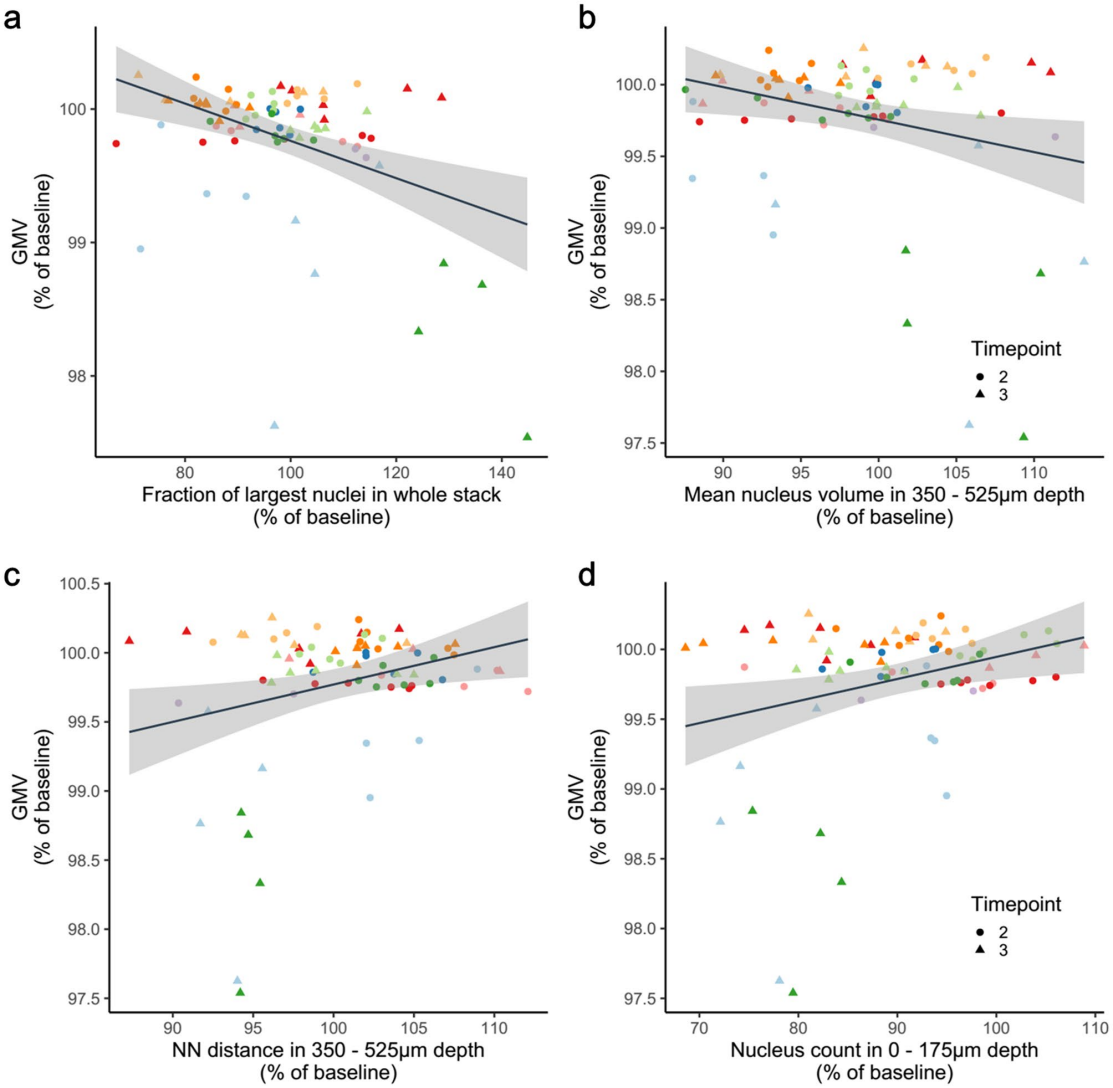
Factors found to significantly influence GMV. At 1 week: n = 45 positions from 9 mice; at 12 weeks: n = 35 positions of 7 mice; Linear mixed models. (**a**) Correlation between the change in fraction of largest nuclei (2250–3000 µm^3^) within the whole stack and the respective JD p = 0.0001, R^2^ = 0.130, Estimate = − 0.013%. (**b**) Correlation between relative change in mean nucleus volume within a depth of 350–525 µm and the JD; p = 0.0005, R^2^ = 0.0964, Estimate = − 0.027%. (**c**) Correlation between nearest neighbour distance changes within a depth of 350–525 µm and the JD. p = 0.002, R^2^ = 0.087, Estimate = 0.030%. (**d**) Correlation between the change in number of nuclei within a depth of 0–175 µm and the respective JD; p = 0.021, R^2^ = 0.044, Estimate = 0.012%.

Finally, we determined the mean volume of all nuclei. With this measure we intended to gain additional information about the identity of the cells corresponding to any given nucleus. A change of observed mean nucleus volumes could hint at a change in the relative abundancy of different cell types within the investigated cortical volumes or a general shrinkage or enlargement of nuclei (see “Discussion”). The average nucleus volume shrank at week 1 (Fig. 7g,h). The details of changes in nucleus sizes observed over time in our study can be inspected in Supplementary Figure 5). Changes in nuclear volume exhibited a significant and inverse correlation with the GMV (Fig. 7i). Hence, a decrease in GMV was accompanied by an increase in mean nuclear volume. Upon this finding, we subdivided all nuclei into four size categories and determined the fraction with which they contributed to the total cell count. When correlating changes in these fractions with GMV, we found the fraction of largest nuclei (between 2250 and 3000 µm^3^) to be the best predictor for GMV, explaining 13% of its variance and proving that a higher proportion of cells with large nuclei is associated with lower GMV (Fig. 8a). This finding could point towards the importance of cell type composition for the interpretation of VBM measurements.

### Factors influencing GMV depend on cortical depth

The findings above were derived from the whole 2Pii stack volumes. Since the cerebral cortex is organized in distinct layers, it seems possible that mechanisms affecting GMV might differ between cortical depths. We addressed this question by subdividing the microscopy stacks in layers of 175 µm thickness along the Z-axis. The layer-confined cellular metrics were correlated with GMV change within the previously defined MRI masks. The effect of mean nucleus volume appeared to be strongest within a depth of 350–525 µm (Fig. 8b). At the same depth, a higher distance between nuclei indicated a larger GMV (Fig. 8c). A positive correlation between nucleus count and GMV was found in the superficial layer (0–175 µm, Fig. 8d). Concluding from these depth-dependent observations, the processes associated with GMV change seem to be composed of varying biological mechanisms that are different throughout cortical layers.

### Combining cellular factors to explain GMV

We next tested if GMV changes can be better described by a model that includes more than one cellular metric. Together, convex hull and the fraction of largest nuclei explained 30.0% of the GMV variance (p-values/estimates of fixed-effect terms: convex hull = 0.019/0.038, fraction of largest nuclei = 0.001/− 0.013 VIF = 1.001). A three-factor model using convex hull, mean nucleus volume in the whole stack and nucleus count in a depth of 350–525 µm achieved an R^2^ of 0.356 (p-valueas/estimates of fixed-effect terms: convex hull =  < 0.001/0.063, mean nucleus volume =  < 0.001/− 0.081, nucleus count = 0.020/0.008, VIF = 3.29/1.20/3.37). These results show that up to 35.6% of the variance in GMV can be explained by a combination of tissue parameters investigated in this study. Main contributing factors are (i) the microscopically measured tissue expansion or shrinkage, (ii) the volume of nuclei, possibly representing cell-type composition, and (iii) cellular density in a depth projected to the transition between cortical layer 2/3 and 5.

## Discussion

This explorative study combined small animal MRI with alternating 2Pii to systematically correlate VBM measures with cellular metrics in vivo. The longitudinal design delivered intra-individual comparisons at different time points in a paradigm based on changes in GMV over a time course of 12 weeks. We found that measured physical tissue volume constituted only a small part of the GMV result, and the average nucleus volume proved to correlate to a similar extent. Restricting the correlations to layers of cellular imaging data revealed that nucleus count and NN distance were correlated with GMV in a layer-specific manner. Nevertheless, even when combined, these contributions do not entirely explain the changes observed in GMV. Taken together, our results suggest that GMV changes are not solely dominated by changes in actual physical volume, but that nuclear volume, local cell number and spatial cell clustering characteristics contribute significantly.

### Technical aspects

Our longitudinal approach exhibits major methodological differences from previous studies which had cross-sectional designs and determined microscopic correlates in fixed brain slices ex vivo^18,21^. The procedure of brain fixation with paraformaldehyde likely affects brain volume and shape^24,25^, with some areas being more affected than others. When performing volumetric morphometry on fixed brains, this artificial distortion in volume might cover up effects actually present in native state or exaggerate false positives. Along these lines, also investigations of histology in fixed brains after conducting VBM in vivo might make correlations invalid. Unwanted effects of brain fixation and preparation can thus only be avoided if both modalities are recorded in the native state, which is also more comparable to the VBM studies on living human subjects.

Despite these considerations, several technical issues linked with the methods used here might limit the conclusions of our study: (i) Compatibility of chronic cranial windows with VBM. The implanted windows, while necessary for high fidelity 2Pii, may affect the MRI signal and thus impact the VBM procedure. We found a change in the physical boundaries of the brain in the frontal area that was improved by using curved windows instead of flat ones, closely following the anatomical shape of the brain (Supplementary Figure 1). In our T2-weighted anatomical MR images we found hyperintense areas close to the edges of the window in some mice that we attribute to CSF accumulation, local edema or scar tissue formation along the rim of the window. On the other hand, these areas were outside of the brain and could be eliminated by the automatic brain extraction algorithm. In summary, we think that these effects do not have a major impact on our VBM analysis, supported by the finding that the GMV changes described here are within the range found in previous studies^14^. (ii) Biological effects of the cranial surgery itself. The open-skull procedure with removal of the dura and implantation of a window was chosen for its superiority over a thinned-skull approach in terms of long-term image quality and achievable imaging depth^26^. This invasive procedure has been shown to trigger changes in underlying brain cells through inflammation^26,27^. To allow the tissue response to subside, we strictly adhered to a recovery period of 4 weeks after surgery, following established protocols^26,28,29^. Dura removal is common practice of in vivo imaging in rats^30–32^ and has been preferred by our and other groups in the past for mouse studies as well^33–36^. When dura removal is performed carefully, glial or astrocytic activation in the underlying tissue can be minimized^31,37^. Despite these considerations, we cannot entirely rule out that the window itself added to the cellular and tissue response that we saw in both MRI or 2Pii over time. The focus of our study, however, is not the exact quantification of the effect of ageing on brain structure, but the correlation of two imaging modalities. Hence, as all mice were treated the same way when imaged with both imaging modalities, the correlations are valid, irrespective of the cause of the underlying tissue change. The effect of time and ageing was used as a test case to obtain measurable changes. (iii) Influence of anaesthesia on brain histology and GMV. It is known that hormonal changes, medication and a number of other different factors can influence global brain volume on a short-time basis^38^. Hence, it is possible that an anesthetic agent similarly affects the tissue during measurements. To rule out a cross-modal bias caused by anesthesia, the same anaesthetic and depth of anaesthesia were applied, hence providing comparable conditions. (iv) Precision of cross-modal image registration. Although all registered masks have been controlled carefully by comparing vessel patterns in both imaging modalities, errors in coregistration cannot be excluded. It also has to be noted that the convex hulls cannot cover the entire mask volume and thus contain varying aspects of the mask volume. This might increase variation in the correlation between GMV and convex hull volume changes. (v) Counting cells in 2Pii imaging volumes. Since all nuclei are reliably labeled in Histone-GFP positive mice (Fig. 4a–c) and counting is carried out automatically without experimenter bias and after exclusion of incomparable image qualities, the cell count within the microscopy images can be considered robust and feasible for comparisons. However, notice should be taken of the fact that the laser cannot penetrate large blood vessels which are found on the cortical surface in some image positions, leading to ‘shadows’ below the vessel with no signal. The number of detected cells in different stack positions can therefore differ to a great extent, and the real total number of cells existent in this region is underestimated. However, this is not a problem for our study, because we always compare the same stack positions and their cell counts longitudinally. (vi) Assessing the volume of nuclei throughout the 2Pii stack. The strength of the histone signal and related to that, measured nucleus volume, is influenced by several parameters such as depth within the tissue and closeness to blood vessels (Supplementary Figure 3). For this reason, again only the changes captured longitudinally represent valid parameters. Any direct inter- or intraindividual comparisons of absolute nucleus volumes between stack positions or layers are problematic and were necessarily avoided in this study.

Taken together, we conclude that the approach introduced here represents a valid and advantageous strategy to identify mechanisms contributing to GMV changes.

### Correlation of GMV with cellular metrics

Our study addressed four structural parameters that may each contribute to a certain extent to GMV changes:

#### Physical tissue volume

The actual change of physical volume within the tissue remains to be validated for the vast majority of cases where VBM has been applied. Keifer et al.^18^ have approached this question comparing GMV with cortical thickness ex vivo in fixed brain slices. They did not find significantly thicker cortices in regions where ex vivo VBM had revealed GMV increases. In our study we introduced a 3-dimensional readout of physical volume changes by taking a defined reference space between reidentified cell nuclei as measurement for local cortical volume. We collected evidence that volumetric measures derived from VBM are indeed accompanied by cortical volume alterations on the microscopic level. However, this relationship turned out to be weaker than expected. In fact, we could show that several cellular parameters significantly add to GMV variance. This observation is in line with the findings of Keifer et al.^18^ in that it further supports the hypothesis that structural plasticity influences GMV readout beyond a solely volumetric mechanism.

#### Cell number

The analysis of cell count revealed a loss of cells in the 2Pii stacks after one week. We assume that one possible cause of this might be the frequent and long periods of deep anaesthesia the mice underwent during the first week of correlative imaging, with approximately one hour of isoflurane anaesthesia for the baseline and 1-week MRI each as well as 4 h for the 2Pii recordings per timepoint. At 12 weeks, the mice seem to have partly recovered from this drop in cell number.

The decrease of cell count did not correlate with changes in GMV when comparing entire imaging volumes. The layered organization of the cortex prompted us to limit correlations to smaller subvolumes corresponding to different cortical layers. With this approach, a significantly higher GMV was observed to be accompanied by a higher cell density in the superficial region from 0 to 175 µm in Z (Fig. 8d). This region mainly consists of the entire cortical layer 1, which is easily distinguishable from deeper layers (Fig. 4f). Although this layer has the sparsest cell density of all, there is an evident complexity and variety of glial cells and inhibitory neurons which have an important influence on local cortical as well as long-range connections in the brain, critical for sensory perception and attention^39–42^. Altering abundance of cells in this connective hub might thus mediate a large impact on structure and function of neighbouring and remote areas. In a more general sense, the proposed role of cell density for VBM results entails consideration of mechanisms leading to cell death, neurogenesis, astrogliosis, and microglial migration. Often, these processes are organized in circumscribed areas, rather than being distributed across entire neocortical layers^43,44^. In further studies, these candidate key cells need to be investigated more directly. For this, the same methods and study design proposed here can be applied to other mouse lines that express fluorescent proteins in specific cell types, using e.g. a GFAP promoter for labeling astrocytes^45^, or a promoter for targeting subtypes of GABA-ergic interneurons (such as Parvalbumin, Somatostatin, VIP or NPY)^46^.

#### Nearest neighbor distance (NN)

When looking at correlations of the NN with GMV within the entire 2Pii volume, no significant relation could be detected. However, when limiting the analysis to deep cortical layers, changes in NN distances were significantly correlated with changes in GMV. If the cells are further away from their next neighbor cells, GMV seems to increase. As cellular density did not predict GMV in this layer, we interpret the NN in this case as a difference in cell proximity without losing or gaining in total cell number; e.g. by a change in the degree of clustering of cells.

#### Nucleus volume

The mean nucleus volume was the only parameter that significantly correlated with GMV when analyzing entire matched 2Pii volumes. Accordingly, the larger the average nucleus volume gets, the smaller the associated GMV becomes. Two processes can lead to changes in average nucleus size: (i) a shift in the relative abundancy of cells harboring nuclei of particularly small or large sizes, as it is known that glial cells have smaller nuclei than neurons; or (ii) a general shrinkage or enlargement of nuclei, which can be a direct indicator for different transcriptional activity states^47^. The subdivision of nuclei into different size categories lead us to the identification of the fraction of largest nuclei as the best predictive factor for GMV in this study. We thus hypothesize that the cell-type composition of cortical tissue influences GMV. Since it is known from brain histology, the largest nuclei in the cortex mostly belong to neurons. It might thus be the relative amount of specific neurons in the cellular architecture that is most influential. This will have to be tested in detail in further studies that include the identification of all cells in the tissue. Biedermann et al.^13^ support a contribution of cellular composition by proving a significant correlation between GMV and the count of DCX-positive cells, a marker for newborn neurons, and GFAP, indicative for astrocytes.

#### Other possible cellular influences on GMV

The multivariable mixed-effect model suggests that the aforementioned parameters might explain approximately 36% of the GMV variance. This leaves space for various other features that were not captured in this study. Previous reports suggested that neuronal structural plasticity may contribute to changes in GMV^13,18,48^. For example, Keifer et al.^18^ proposed that a higher spine density could explain 20% of the increased ‘VBM signal’ in the auditory cortex of mice after auditory fear conditioning compared to a control group^18^, yet this spine plasticity did not result in thicker cortices. Here, we did not directly investigate the contribution of spine dynamics, but our results are compatible with these previous findings. Possible processes interpreted as volume change by VBM, but missed by our metrics, might be caused by changes in regional blood perfusion or dehydration^20^, with accompanying effects ranging from differences in diffusion to changes in T1 or T2 relaxation. Furthermore, since the coregistration of MR images, from which the JD is obtained, is mostly directed by rather coarse tissue boundaries consisting of pial surface and the boundary between gray and white matter, it is plausible to assume that a certain extent of the variability in MRI that could not be explained by our readouts it due to processes that are beyond reach of the microscopy we used. We attempted to circumvent this problem by choosing more shallow MRI masks (see “Methods”), but this possible confound can not be entirely ruled out.

### Age-dependent changes in VBM and underlying cellular mechanisms

The gray matter changes detected by VBM follow a pattern previously described^14^, with age-related increases in the cerebellum and subcortical areas (midbrain, hindbrain, thalamus, hypothalamus and pallidum/lateral septum), and reduced GMV gain or loss of volume being present in the olfactory bulb and distinct cortical areas (visual cortex, somatosensory area and motor cortex as well as parts of the anterior cingulate). At week one, a temporary lateralization emerged, with a trend for GMV loss in the right and gain in the left frontal cortex (p = 0.063, cluster corrected). Cerebrocortical asymmetry in rodents have been shown to be dictated by age, but also environmental factors and gender^49–52^. Our findings replicate previous reports by Spring and colleagues, who demonstrate smaller right motor and sensorimotor cortex in comparison to the left side in 12 week-old C57Bl/6 mice^53^. Yet, the dynamics at which age-related asymmetries come about remain unclear. To elucidate the nature of such structural asymmetries, further studies comparing left and right hemisphere also on the cellular level are needed. The extent of increase in overall GMV that we measured was found to be lower than in previous studies^14^, a finding which might be attributed to the older starting age at first MR imaging in the present study. At the timepoint of the first imaging, mice were 3 months old, corresponding to a human age of about 20 years. At the latest timepoint studied here, mice were 6 months old and hence in their mature adulthood, roughly corresponding to a 30 year old human^54^. Overall, the described changes are consistent with those found in human aging^5^, underlining the cross-species validity of the present approach for the recapitulation of age-related changes.

On the cellular level, myelination in upper cortical layers decreased while it increased in lower cortical layers at 3–6 months of age^55^, consistent with our finding of increased nearest neighbour distances and a higher GMV in deeper layers. Less spine plasticity in older mice^56^ may reduce the space between reidentified cells and hence may contribute to the general reduction in nearest neighbor distances. The single best factor explaining variance in GMV signals was the fraction of large nuclei within the imaging region, consistent with a significant decline of oligodendrocyte precursor cells, neuronal-restricted precursors and immature neurons as well as a trend towards a reduction in certain astrocyte subtypes during aging^57^. These cells all have smaller nuclei and therefore may explain the increase of the fraction of large (neuronal) nuclei and decrease in GMV described here during aging of the cerebral cortex.

The approach presented here is principally suited to further characterize mechanisms of brain atrophy during senescence, but problems such as higher drop-out rates during anesthesia for surgery and imaging sessions may need to be considered in older mice (18–24 months)^58^. Longitudinal imaging studies with 2Pii in developing animals (age 0–8 weeks) are unlikely to work using cranial windows owing to the continued growth of the skull, yet the thinned skull approach^59,60^ may provide a suitable alternative.

### Statistical considerations: group effects versus longitudinal correlations within individuals

Our work can be separated into (i) the descriptive analyses of putative effects of time on the measured parameters (GMV, cell count, NN distance, nucleus volume) and (ii) the intermodal correlation of GMV changes with cellular parameters from microscopy (see “Methods” section, “Statistical information”). It is important to note that these two analyses are independent from each other. In this study, ageing was the vehicle to produce GMV change on an individual, not a group level. We put a focus on finding general concepts that can explain GMV change on a cellular basis rather than describing the biology of ageing. Even if the mean of the GMV was statistically completely identical on a group level at the different timepoints, the changes for each individuum can still be highly correlated with the cellular 2P changes. One could in principle study correlations from GMV and cell number, for example, on only one single timepoint, without having any groups to compare on a descriptive level. But taking the cellular parameters on a single timepoint entails, as explained in detail above, major drawbacks in terms of inter- and intraindividual comparability in the methods we used. To avoid this and to increase the power of our study, we decided to use a longitudinal design and to look at the *change* in cellular parameters and GMV, allowing us to normalize every parameter to the individual’s baseline. This indicates that, irrespective of group effects, the correlations of registered parameters stay valid and could thus also represent a general mechanism, which might not (only) be related to the investigated condition.

## Conclusion

This study provides a systematic assessment of several structural parameters and their contribution to changes in GMV in the living mouse brain. Our data show that GMV changes are not strongly related to changes in physical volume, suggesting that the results of VBM cannot solely be interpreted as mere changes in tissue volume. Of the many possible mechanisms within the tissue that may influence the GMV, our work proposes that local cell count, spatial arrangement of cells as well as cell type composition are factors that make important contributions. The results suggest that spatially restricted, layer-specific processes may differentially influence GMV within a cortical region of interest. Our explorative, nucleus-imaging-based approach thus indicates a multicausal relationship of tissue characteristics and GMV, and provides a basis for studies focusing more on the abundance, distribution and morphology of specific cell types including neurons, glia and vasculature, along with their subcellular compartments (dendrites, spines, processes, etc.). The analyses provided here are all of correlative nature, causality would have to be probed with approaches that manipulate cellular factors and subsequently probe GMV changes. We conclude that the investigation of structural parameters with correlative in vivo imaging is substantial for the open quest to understanding the cellular mechanisms underlying voxel-based morphometry.

## Materials and methods

### Ethical approval

This study was carried out in accordance with the European Communities Council Directive (86/609/EEC) to minimize animal pain or discomfort. All experiments were conducted following the German animal welfare guidelines specified in the TierSchG. The local animal care and use committee (Regierungspräsidium Karlsruhe of the state Baden-Wuerttemberg) approved the study under the reference number G294/15. All experiments complied with the ARRIVE guidelines^61^.

### Mice

Adult transgenic mice of C57BL/6 background expressing enhanced green-fluorescent protein fused to human histone H2B (B6.Cg-Tg(HIST1H2BB/EGFP)1 Pa/J, RRID:IMSR_JAX:006069), short ‘Histone-GFP’^62^, were purchased from Jackson Laboratories (stock #006069) and bred in our animal facility at IBF (Interfakultaere Biomedizinische Forschungseinrichtung). Since the transgene is controlled by a ubiquitously active CAG-promoter, nuclei of all cells express the Histone-GFP fusion protein and can be identified by fluorescence imaging^62^. After cranial window surgery, mice were housed separately in individually ventilated cages at a 12-h light–dark cycle and in a temperature- (22 ± 2 °C) and humidity- (60 ± 4%) controlled environment. Food and water were available ad libitum. Twelve mice divided into four batches of three mice each entered the study. Animals within a batch were littermates. Mice of either sex were chosen at equal numbers. Four animals could not complete the trial and had to be sacrificed at varying timepoints due to window detachment, bleeding, infection or poor image quality in 2Pii.

### Experimental design

Chronic cranial windows were implanted in mice at 8 weeks of age. Baseline MRI was acquired 4 weeks after window surgery and followed by 2Pii one to two days later. Six 2Pii imaging positions were recorded in each animal per timepoint, arranged in a line from rostral to occipital to cover large parts of the anterior- and midcingulate and motor cortices. MRI and 2Pii measurements were repeated at 1 week and at 12 weeks after baseline. All sessions were carried out at consistent times of day. For a visual display of the experimental timeline see Fig. 1b. In the investigated set of mice, we conducted a sham surgery as part of the spared nerve injury model^63^ on the day after baseline measurements, because these animals served as controls in a study related to neuropathic pain. The procedure included a 10 min surgery on the left hind limb under isoflurane anaesthesia with skin incision, separation of muscles and exposure of the sciatic nerve branches without further intervention before closing the skin.

### Curving glass windows for to match natural brain curvature

The mouse skull exhibits a curvature on the mediolateral axis while it is more straight in the rostrocaudal axis. For the cranial window to adapt most closely and avoid regional flattening of brain tissue, we bent glass windows along one diameter axis. Round glass coverslips of 6 mm diameter and 100 µm thickness (VWR, Thickness No. 0, Cat.-No. 631-0168) were placed in row in a custom-cut graphite mold groove (graphite from CGC Klein, material CG 1290, Siegen, Germany). A cylinder of 20 mm diameter matching into that mold was carefully placed on top of the glasses. Graphite and glasses were heated at 600 °C in a glass tempering furnace for 15 min to allow the weight of the graphite cylinder to shape the heatened glass (Supplementary Figure 1)^64^.

### Chronic cranial window surgery

Cranial window surgery was performed as described elsewhere^35^. Briefly, mice were anesthetized by intraperitoneal injection of a narcotic mix consisting of 120 µl Medetomidine (1 mg/ml, Sedin), 320 µl Midazolam (5 mg/ml, Hameln) and 80 µl Fentanyl (0.05 mg/ml, Janssen) at a dosage of 3 µl per gram body weight. A craniectomy (diameter 6 mm) centered 1 mm rostral to bregma was created with a dental drill. The dura was removed carefully with fine forceps on the exposed part of the right hemisphere to ensure long lasting high image quality. Curved coverslips and a custom-made light-weight plastic holder ring were positioned on top of the exposed area and cemented to the surrounding skull with dental acrylic, cautiously preventing any glue from touching the brain surface.. For mice to recover from anesthesia after completing the procedure, a mix containing 120 µl Atipamezole (5 mg/ml, Prodivet), 120 µl Flumazenil (0.1 mg/m, Fresenius Kabi) and 720 µl Naloxon (0.4 mg/ml, Inresa) was injected intraperitoneally and subcutaneously at 3 µl per gram body weight each. To relieve postsurgical pain, 250 µl of Carprofen (50 mg/ml, Carprieve) was injected subcutaneously immediately after surgery and given every 8–12 h until 24 h after the surgery. Mice were then placed in their home cage placed on a heating plate and monitored until normal locomotion and grooming behaviour was observed.

### MRI

MR data were acquired in a 9.4 T horizontal bore animal scanner (Bruker, Ettlingen, Germany) with a two-element anatomically shaped cryogenic mouse surface coil cooled to 28 K. The cryogenic coil gives an improvement factor of 2.5–3.5 in signal-to-noise ratio compared to the conventional setup of a 4-channel receiver array and a volume transmit coil. The animals were anaesthetized by a gas mixture of O_2_: 30% and air: 70% with 2% Isoflurane during each measurement. Respiration rate and body temperature (maintained at 36 °C) were monitored throughout the experiment. High-resolution 3D structural images were acquired with a T2-weighted RARE sequence (Rapid Acquisition with Refocused Echoes, RARE factor 16, matrix size 225 × 192 × 96) with an echo time TE = 50 ms, a repetition time TR = 1.2 s and a spatial resolution of 78 µm × 78 µm × 156 µm. Slice orientation: read-Out direction in anterior/posterior; phase encode direction: left/right. Slice direction in superior/inferior direction. The acquisition bandwidth was 7500 Hz. Neither parallel imaging nor partial Fourier were used. We measured the T2 relaxation times in the prefrontal cortex and cingulate cortex with a multi-spin-multi-echo (MSME) sequence which resulted in cortical T2 = 45 ms. The changes of the point spread function (PSF) occurring in the phase encode direction was calculated^65^:

$$FWHM_{trunc + decay} = 1.23 - 0.05\left( {\frac{{T_{k} }}{{T_{2} }}} \right) + 0.08\left( {\frac{{T_{k} }}{{T_{2} }}} \right)^{2} .$$

With an echo spacing of 6.25 ms and the trajectory time T_k_ = 16 × 6.25 ms = 100 ms, we calculated a PSF-blurring of a factor of 1.514 of the nominal voxel-size in left–right direction.

### Longitudinal VBM analysis

All MRI data was processed with MATLAB 9.2 (The MathWorks, Inc., RRID:SCR_001622) using SPM12 (https://www.fil.ion.ucl.ac.uk/spm/software/spm12, RRID:SCR_007037) and customized MATLAB code. Image pre-processing was similar to longitudinal human studies^66^, further details can be found in the Supplementary Material. In short: The acquired structural images were resized by a factor of 10 (only the matrix defining the image position in space was changed for better SPM compatibility) and registered to a template^13^ in Paxinos standard space (rigid body registration without data reslicing). We analysed brain volume changes between baseline and 1 week as well as baseline and 12 weeks separately in several steps.

1. Pairwise longitudinal non-linear registration was performed for each subject with SPM12 using the 1 week and 12 weeks—timepoints in comparison to baseline in order to analyze structural changes^67^. This procedure resulted in three distinct images for each data pair: an average image of the two analyzed time points for each subject, a vector field depicting the local shift of each voxel for the registration and an image of the Jacobian rate describing the local volume change in each voxel between the two time points (Fig. 2c) with values below zero depicting volume loss and values greater than zero depicting gain between two analysed timepoints.
2. Average images were skull-stripped and segmented, separating gray matter, white matter and cerebrospinal fluid using a Gaussian mixed model and tissue probability masks from previous work^13^. The tissue segmentation serves two purposes: (i) to create a group-specific Dartel template and normalize all data into template space and (ii) to weigh the Jacobian rate images by the individual tissue maps.
3. The Jacobian rate of difference images from step (1) were multiplied by the GM image from step (2) to derive a GMV rate of change map (GMV-C, Fig. 2c, middle column).
4. The unmodulated tissue images were entered into the DARTEL toolbox (SPM)^68^ to create a group-specific tissue class template and subsequently to normalize all GMV-C images to a common Paxinos space using the flow fields that are a result of the DARTEL procedure.

The normalized GMV-C images, depicting the volume difference between two time points (0 vs. 1 week and 0 vs. 12 weeks) were smoothed (4 mm Gaussian kernel) and statistically tested in a second-level (*t* test) analysis as well as with a non parametric TFCE (http://dbm.neuro.uni-jena.de/tfce) with 5000 permutations. While the Jacobian rate is the driving mechanism for the detection of longitudinal structural differences within subject changes, segmentation into tissue classes accounts mainly for between-subject difference in local GM distribution. For the regional analyses based on selected atlas regions, the individual normalized GMV-C images were overlaid with a digital mouse atlas and the values for selected regions were extracted and analysed with one sample *t* test. The values depict volume change in percent of baseline volume.

The tissue segmentation was used only for the whole brain analyses of the VBM data, but not for the 2Pii mask statistics. Instead, for the analysis of volume changes over time within the 2Pii voxels, values from the Jacobian rate images corresponding to each individual 2Pii mask were extracted by transforming the mask images into the average space of each subject. Mean Jacobian rate values were extracted slicewise (dorsoventral direction) for each mask and used for correlation analyses.

### Cross-modal image registration

To define exact regions of interest that represent the volumes of every single 2Pii image stack in the respective MRI space, we exploited superficial venous vessel branching points as natural fiducial landmarks visible in both imaging modalities (Fig. 2a). An individual 2-dimensional affine registration for the XY-positions of the 2Pii image volumes was inferred on the MR image for every animal using 3 vessel branching points as fiducials. Z-positions and tilts around all axes were calculated and binary masks fitting the 2Pii-stack dimensions inside the MRI volume were created (Fig. 2b). For an enlarged view of the location of 2Pii masks in the MRI volume, see also Supplementary Figure 6. The Z-depth of these MRI masks include only the upper three voxel planes in Z, limiting GMV calculations to a depth of about 450 µm from the brain surface instead of the full 700 µm as it is captured by 2Pii. The rationale for this stems from the fact that coregistration and smoothing during VBM cause spatial blurring of results, leading to influences from of brain areas situated below the reach of 2Pii on the measurmentes within the MRI masks. By limiting the MRI masks to the superficial cortex we aimed to minimize these possible confounds. The binary masks were then transferred to the average image of two compared time points by applying vector fields resulting from longitudinal nonlinear registration and GMV was determined within each of these masks,

### Two-photon in vivo imaging (2Pii)

Inhalation anesthesia was initiated with 4–5% Isoflurane (Baxter) and adjusted according to respiration rate at about 0.5–2% with a O_2_-flow rate of 0.5 l/min. Body temperature was maintained via heating plate. Eye ointment was applied and the head was tightly fixated with two metal bars enclosing the head ring to avoid movement artifacts. Two-Photon imaging was conducted with a TriM Scope II microscope (LaVision BioTec GmbH) equipped with a pulsed Ti:Sapphire laser (Chameleon; Coherent) at an excitation wavelength of 960 nm. Emitted fluorescence was passed through a 530/70 nm band pass filter (Chroma) before being collected and amplified a by low-noise high-sensitivity photomultiplier tube (PMT; Hamamatsu, H7422-40-LV 5M). A 16 × objective with 0.8 numerical aperture, 3 mm working distance and water immersion (Nikon) was used to scan 3D image stacks with a field of view of 700 µm × 700 µm in XY until a depth of 700 µm below the cortex surface was reached. Image planes were sampled at a step size of 2 µm (voxel size 0.29 µm × 0.29 µm × 2 µm). PMT noise offset was checked and corrected prior to each imaging session to be stable throughout the whole experiment. Increase of laser power was set in defined depths for every mouse individually to assure good signal to noise image acquisition while minimising the number of oversaturating pixels. Six stack positions were recorded in a straight line immediately lateral to the superficial sagittal sinus from rostral to occipital (with a 15% inter-stack- overlap, Fig. 2c), covering large parts of the anterior- and midcingulate cortex and primary and secondary motor areas. Stack positions were reidentified at later time points by superficial vessel orientation and aligning image position by nuclei patterns in a depth of ~ 200–250 µm below the cortical surface.

### Confocal microscopy of fixed brain slices

Mice were deeply anaesthetized with intraperitoneal Narcoren (500 mg/kg bodyweight) and transcardially perfused with 4% paraformaldehyde (PFA) in phosphate‐buffered saline (PBS). The brains were post-fixed overnight in 4% PFA. After washing with PBS, 50 µm thick coronal brain sections were cut with a vibratome. The slices were incubated separately in wells with PBS and 5 ng/ml DAPI (4′,6-diamidino-2-phenylindole) for 20 min before they were rinsed in PBS and mounted in SlowFade Gold (Life Technologies). Superficial cortical areas of anterior and midcingulate cortex were imaged on a Leica SP8 inverted confocal microscope with a 63 × oil immersion objective (numerical aperture = 1.4) and a resolution of 0.06 μm × 0.06 μm × 0.76 μm. Samples were illuminated with a 405 nm laser for DAPI and 488 nm for Histone-GFP in sequential scans. Detectors collected light from 409–464 nm for DAPI (PMT) and 491-564 nm for Histone-GFP (Leica HyD).

### Analysis of physical tissue volume changes

To analyze regional cortical volume changes, we identified nuclei patterns in the image stacks that were stable over time, indicating that a nucleus within this pattern did not individually migrate and thus serves as a fiducial marker for a stable position within the stack (Fig. 5a). These marker nuclei were sampled throughout the whole stack (> 30 to obtain reliable results). Every change of a volume spanned between markers were consequentially interpreted as an expansion or shrinkage of the tissue volume in between them.

For this analysis, three of the six acquired 2Pii stacks were chosen for every animal at random to probe for general GMV correlations in varying regions. Stack coordinates of the centres of re-identified marker nuclei were collected for every timepoint manually in these stacks using FIJI (RRID:SCR_002285) (Fig. 5b)^69^. From these coordinates, we calculated a 3D Delaunay triangulation in MATLAB and determined the volume formed by the resulting convex hull (Fig. 5c). The convex hull volume could then be readily compared over all three timepoints and is referred to as the readout for physical volume changes in this study. The set of marker nuclei was arbitrarily chosen and sampled to include as much of the total image stack volume as possible. Consequently, the absolute convex hull volume is individual for each image position and, as such, is only suited for interpretation of relative volume changes over time rather than direct comparison between individuals. For layer-confined measures of physical volume change, volumes of tetrahedra within the Delaunay triangulation, whose centroids lie in the respective defined Z-depth, were summed up and compared between timepoints. All computations were done with MATLAB (The MathWorks, Inc., RRID:SCR_001622).

### Automated image analysis

The bioimage analysis pipeline was designed to segment Histone-GFP positive nuclei from 2Pii stacks in 3D (Supplementary Figure 2). The analysis workflow was developed combining different software tools as described below.

#### Image preprocessing

The 2Pii raw images were deconvolved with Huygens SVI software (RRID:SCR_014237) using the CMLE algorithm, with a signal-to-noise-ratio of 7 and 500 iterations (Scientific Volume Imaging, The Netherlands, http://svi.nl). To further improve nucleus detection, we used the machine-learning-based autocontext workflow implemented in ilastik (RRID:SCR_015246)^70,71^. To enhance differentiation from image foreground and background we trained the ilastik classifier to recognize varying signal intensities and textures of nuclei as foreground, providing a foreground probability map^71^. All stacks were processed using ilastik’s batch processing in headless mode executed on the bwHPC high performance computing cluster (see Acknowledgements).

#### 3D nucleus detection and segmentation

Analysis steps of both FIJI and MATLAB were combined in one fully automated custom-written script by using the ImageJ-MATLAB extension plugin^72^. The workflow involved two major steps: 3D seed detection and 3D watershed segmentation. First, seeds were detected on probability maps using FIJI’s 2D ‘find maxima’, dilated using the ‘3D dilate’ operators and linked in 3D when connected to each other in z. Secondly, the 3D seeds and probability maps were used as input for the 3D watershed algorithm. The intensity threshold and assumed nucleus radius required for this operation where chosen carefully to optimize the output of the automated segmentation to match 3D manually segmented ground truth data. False positive segmentations were reduced by filtering out objects with a volume below 1500 voxels. Objects above 18,000 voxels, which suggest wrongly merged or undersegmented nuclei, were re-segmented using a lower 3D watershed threshold value. The output image showed individually labeled objects representing seperate nuclei. The ImageJ-Fiji/MATLAB script was used to process all image stacks on a Dell PowerEdge T630 server with 20 cores, 1 TB of RAM with an average running time of 3 h per z-stack. For each input stack, an output folder was created to save 3D-segmented nuclei as labeled objects and a csv file with the xyz nuclei centroid coordinates and the nuclei volume size.

#### Deriving cellular metrics from the segmentation images

Center coordinates (centroids) and volume of all segmented nuclei were calculated in MATLAB and stored in a SQLite Database (RRID:SCR_017672) for every stack. Cell density was assessed by counting all detected nuclei within the whole stack or a certain Z-depth. Distances from centroids of all segmented nuclei to the next neighboring centroid were determined using the spatstat package in R^73^ (RRID:SCR_001905)^74^.

#### 2Pii data exclusion criteria

We identified three causes for incomparable image quality between timepoints: (i) software failure during image acquisition in form of laser blankouts (Supplementary Figure 4c); (ii) change in homogeneity of signal yield throughout the image plane. This is attributable to localized dural regrowth between timepoints or air bubbles forming in the immersion water unnoticedly during acquisition. These were checked for by comparing image frames at a depth of 300 µm and are easily identified by eye (Supplementary Figure 4a); (iii) discrepancies in Signal to noise ratio (SNR). We defined SNRs in the images by measuring pixel intensities of matching image regions of the compared timepoints at a depth of 300 µm. SNR was then defined as.

$$SNR = \frac{{\mu_{sig} }}{{\sigma_{bg} }},$$

where $\mu_{sig}$ is the mean pixel intensity in a reference area with signal (nuclei), and $\sigma_{bg}$ is the standard deviation of pixel intensity in a reference background area (Supplementary Figure 4d). The SNR was rated ‘stable’, when compared SNRs of at least 3 reference areas did not differ more than 10% to ensure robust foreground classification in ilastik.

If one of the exclusion criteria was met, the dataset was excluded from statistical analysis. To achieve an unbiased preselection of stacks that indicates potential incomparability of image quality over time, we established the detected cell density distributions along the z-axis as a surrogate parameter. To compute the density profile of all nuclei, the “density” function from the ‘stats’ package from R^73^ was used Supplementary Figure 4b). When the value of this sum exceeded a certain threshold, we continued examining the respective dataset for the three incomparability causes as listed above. This threshold was set to the value of 500 after a random inspection of stacks with a value below 500 consistently fulfilled none of the exclusion criteria.

### Statistical information

Statistical analysis was performed in R^57^ (RRID:SCR_001905) and Graphpad Prism (RRID:SCR_002798). Linear mixed-effect models were applied to account for multivariate hierarchical structure of our data (six imaging positions are nested within one individual animal) by introducing a random effect for the Animal ID. Models were fit by restricted maximum likelihood and the following formulae were applied:

1. Parameter ~ Timepoint + (1 | Animal-ID)
2. GMV ~ Parameter1 + Parameter2 + Parameter3 + (1 | Animal-ID)

Formula (1) tests the fixed effect of timepoint on single parameters (Figs. 6b,c, 7a,b,d,e,g,h). The term ‘(1 | Animal-ID)’ expresses the random effect on the model intercept in each animal. To test for significant fixed effects of one, two or three 2Pii parameters on GMV change, formula (2) was used (Figs. 6d, 7c,f,i, 8a–d). The variation inflation factors (VIF) are stated in figure legends for all variables of multivariable analyses to avoid multicollinearity. ANOVA, *t* tests or respective non-parametric tests were used when linear mixed-effect models did not fit. Tests were performed two-sided and all distributions were tested for normality prior to choosing the appropriate statistic test. Boxplots highlight the mean, lower and upper hinges correspond to the first and third quartiles, whiskers mark min and max values limited to 2.5 times the first or third quartile respectively. Correlation plots show black regression lines with 95% confidence band in gray. *SD *standard deviation, *F *F value, *DF *degrees of freedom.

## Supplementary information

Supplementary Video 1.

Supplementary Figure 1.

Supplementary Figure 2.

Supplementary Figure 3.

Supplementary Figure 4.

Supplementary Figure 5.

Supplementary Figure 6.

Supplementary Information.

## Data Availability

Comprehensive access to the source data of this study is provided by making it directly available on the heiDATA repository. It includes (i) spreadsheets of data values for each plot in the main figures, (ii) all raw data from 2Pii, (iii) custom written code, (iv) a representative subsets of microscopy raw data and matching segmentation label images, coordinates of reidentified nuclei and corresponding magnetic resonance images from all timepoints. The data is available via the following link: https://doi.org/10.11588/data/04HPWW.

## References

[1] Radua J, Canales-Rodriguez EJ, Pomarol-Clotet E, Salvador R (2014). Validity of modulation and optimal settings for advanced voxel-based morphometry. NeuroImage.

[2] Ashburner J, Friston KJ (2000). Voxel-based morphometry—The methods. NeuroImage.

[3] Salmenpera TM, Duncan JS (2005). Imaging in epilepsy. J. Neurol. Neurosurg. Psychiatry.

[4] May A, Gaser C (2006). Magnetic resonance-based morphometry: A window into structural plasticity of the brain. Curr. Opin. Neurol..

[5] Good CD (2001). A voxel-based morphometric study of ageing in 465 normal adult human brains. NeuroImage.

[6] Grieve SM, Korgaonkar MS, Koslow SH, Gordon E, Williams LM (2013). Widespread reductions in gray matter volume in depression. NeuroImage Clin..

[7] Matsuo K (2019). Distinctive neuroanatomical substrates for depression in bipolar disorder versus major depressive disorder. Cereb. Cortex.

[8] Uchida RR (2008). Regional gray matter abnormalities in panic disorder: A voxel-based morphometry study. Psychiatry Res..

[9] Kuhn S, Gallinat J (2013). Gray matter correlates of posttraumatic stress disorder: A quantitative meta-analysis. Biol. Psychiatry.

[10] Apkarian AV (2004). Chronic back pain is associated with decreased prefrontal and thalamic gray matter density. J. Neurosci..

[11] Matsuda H (2013). Voxel-based morphometry of brain MRI in normal aging and Alzheimer's disease. Aging Dis..

[12] Seminowicz DA (2009). MRI structural brain changes associated with sensory and emotional function in a rat model of long-term neuropathic pain. Neuroimage.

[13] Biedermann S (2012). In vivo voxel based morphometry: Detection of increased hippocampal volume and decreased glutamate levels in exercising mice. NeuroImage.

[14] Bilbao A (2018). Longitudinal structural and functional brain network alterations in a mouse model of neuropathic pain. Neuroscience.

[15] Kuner R, Flor H (2017). Structural plasticity and reorganisation in chronic pain. Nat. Rev. Neurosci..

[16] Pomares FB (2017). Histological underpinnings of grey matter changes in fibromyalgia investigated using multimodal brain imaging. J. Neurosci..

[17] Henderson LA, Di Pietro F (2016). How do neuroanatomical changes in individuals with chronic pain result in the constant perception of pain?. Pain Manag..

[18] Keifer OP (2015). Voxel-based morphometry predicts shifts in dendritic spine density and morphology with auditory fear conditioning. Nat. Commun..

[19] Lerch JP (2011). Maze training in mice induces MRI-detectable brain shape changes specific to the type of learning. NeuroImage.

[20] Streitburger DP (2012). Investigating structural brain changes of dehydration using voxel-based morphometry. PLoS ONE.

[21] Biedermann SV (2016). The hippocampus and exercise: Histological correlates of MR-detected volume changes. Brain Struct. Funct..

[22] Helmchen F, Denk W (2005). Deep tissue two-photon microscopy. Nat. Methods.

[23] Dorr AE, Lerch JP, Spring S, Kabani N, Henkelman RM (2008). High resolution three-dimensional brain atlas using an average magnetic resonance image of 40 adult C57Bl/6J mice. Neuroimage.

[24] Hikishima K (2017). In vivo microscopic voxel-based morphometry with a brain template to characterize strain-specific structures in the mouse brain. Sci. Rep..

[25] Wehrl HF (2015). Assessment of murine brain tissue shrinkage caused by different histological fixatives using magnetic resonance and computed tomography imaging. Histol. Histopathol..

[26] Dorand RD, Barkauskas DS, Evans TA, Petrosiute A, Huang AY (2014). Comparison of intravital thinned skull and cranial window approaches to study CNS immunobiology in the mouse cortex. Intravital.

[27] Xu HT, Pan F, Yang G, Gan WB (2007). Choice of cranial window type for in vivo imaging affects dendritic spine turnover in the cortex. Nat. Neurosci..

[28] Holtmaat A (2009). Long-term, high-resolution imaging in the mouse neocortex through a chronic cranial window. Nat. Protoc..

[29] Lee WC (2008). A dynamic zone defines interneuron remodeling in the adult neocortex. Proc. Natl. Acad. Sci. U.S.A..

[30] Heo C (2016). A soft, transparent, freely accessible cranial window for chronic imaging and electrophysiology. Sci. Rep..

[31] Koletar MM, Dorr A, Brown ME, McLaurin J, Stefanovic B (2019). Refinement of a chronic cranial window implant in the rat for longitudinal in vivo two-photon fluorescence microscopy of neurovascular function. Sci. Rep..

[32] Masuda H, Ushiyama A, Hirota S, Lawlor GF, Ohkubo C (2007). Long-term observation of pial microcirculatory parameters using an implanted cranial window method in the rat. In Vivo.

[33] Andermann ML, Kerlin AM, Roumis DK, Glickfeld LL, Reid RC (2011). Functional specialization of mouse higher visual cortical areas. Neuron.

[34] Boffi JC, Knabbe J, Kaiser M, Kuner T (2018). KCC2-dependent steady-state intracellular chloride concentration and pH in cortical layer 2/3 neurons of anesthetized and awake mice. Front. Cell. Neurosci..

[35] Knabbe J, Nassal JP, Verhage M, Kuner T (2018). Secretory vesicle trafficking in awake and anaesthetized mice: Differential speeds in axons versus synapses. J. Physiol..

[36] Zuluaga-Ramirez V, Rom S, Persidsky Y (2015). Craniula: A cranial window technique for prolonged imaging of brain surface vasculature with simultaneous adjacent intracerebral injection. Fluids Barriers CNS.

[37] Goldey GJ (2014). Removable cranial windows for long-term imaging in awake mice. Nat. Protoc..

[38] Dieleman N, Koek HL, Hendrikse J (2017). Short-term mechanisms influencing volumetric brain dynamics. NeuroImage Clin..

[39] Lanjakornsiripan D (2018). Layer-specific morphological and molecular differences in neocortical astrocytes and their dependence on neuronal layers. Nat. Commun..

[40] Jiang X, Wang G, Lee AJ, Stornetta RL, Zhu JJ (2013). The organization of two new cortical interneuronal circuits. Nat. Neurosci..

[41] Lam YW, Sherman SM (2019). Convergent synaptic inputs to layer 1 cells of mouse cortex. Eur. J. Neurosci..

[42] Vogt BA (1991). Normal and Altered States of Function.

[43] Cotter D (2002). Reduced neuronal size and glial cell density in area 9 of the dorsolateral prefrontal cortex in subjects with major depressive disorder. Cereb. Cortex.

[44] Lemmens MA (2011). Age-related changes of neuron numbers in the frontal cortex of a transgenic mouse model of Alzheimer's disease. Brain Struct. Funct..

[45] Nolte C (2001). GFAP promoter-controlled EGFP-expressing transgenic mice: A tool to visualize astrocytes and astrogliosis in living brain tissue. Glia.

[46] Mehta P (2019). Functional access to neuron subclasses in rodent and primate forebrain. Cell Rep..

[47] Fair T, Hyttel P, Greve T (1995). Bovine oocyte diameter in relation to maturational competence and transcriptional activity. Mol. Reprod. Dev..

[48] Suzuki H (2013). Voxel-based morphometry and histological analysis for evaluating hippocampal damage in a rat model of cardiopulmonary resuscitation. NeuroImage.

[49] Diamond MC, Johnson RE, Young D, Singh SS (1983). Age-related morphologic differences in the rat cerebral cortex and hippocampus: Male–female; right–left. Exp. Neurol..

[50] Dowling GA, Diamond MC, Murphy GM, Johnson RE (1982). A morphological study of male rat cerebral cortical asymmetry. Exp. Neurol..

[51] Zilles K (1996). Structural asymmetries in the human forebrain and the forebrain of non-human primates and rats. Neurosci. Biobehav. Rev..

[52] Bulman-Fleming B, Wainwright PE, Collins RL (1992). The effects of early experience on callosal development and functional lateralization in pigmented BALB/c mice. Behav. Brain Res..

[53] Spring S, Lerch JP, Wetzel MK, Evans AC, Henkelman RM (2010). Cerebral asymmetries in 12-week-old C57Bl/6J mice measured by magnetic resonance imaging. Neuroimage.

[54] Fox JG (2007). The Mouse in Biomedical Research.

[55] Hammelrath L (2016). Morphological maturation of the mouse brain: An in vivo MRI and histology investigation. NeuroImage.

[56] Runge K, Cardoso C, de Chevigny A (2020). Dendritic spine plasticity: Function and mechanisms. Front. Synaptic Neurosci..

[57] Ximerakis M (2019). Single-cell transcriptomic profiling of the aging mouse brain. Nat. Neurosci..

[58] Schuetze S, Manig A, Ribes S, Nau R (2019). Aged mice show an increased mortality after anesthesia with a standard dose of ketamine/xylazine. Lab. Anim. Res..

[59] Drew PJ (2010). Chronic optical access through a polished and reinforced thinned skull. Nat. Methods.

[60] Yang G, Pan F, Parkhurst CN, Grutzendler J, Gan WB (2010). Thinned-skull cranial window technique for long-term imaging of the cortex in live mice. Nat. Protoc..

[61] Percie du Sert N (2020). The ARRIVE guidelines 2.0: Updated guidelines for reporting animal research. J. Physiol..

[62] Hadjantonakis AK, Papaioannou VE (2004). Dynamic in vivo imaging and cell tracking using a histone fluorescent protein fusion in mice. BMC Biotechnol..

[63] Decosterd I, Woolf CJ (2000). Spared nerve injury: An animal model of persistent peripheral neuropathic pain. Pain.

[64] Kim TH (2016). Long-term optical access to an estimated one million neurons in the live mouse cortex. Cell Rep..

[65] Qin Q (2012). Point spread functions of the T2 decay in k-space trajectories with long echo train. Magn. Reson. Imaging.

[66] Meda SA (2017). Heavy drinking in college students is associated with accelerated gray matter volumetric decline over a 2 year period. Front. Behav. Neurosci..

[67] Ashburner J, Ridgway GR (2012). Symmetric diffeomorphic modeling of longitudinal structural MRI. Front. Neurosci..

[68] Ashburner J (2007). A fast diffeomorphic image registration algorithm. NeuroImage.

[69] Schindelin J (2012). Fiji: An open-source platform for biological-image analysis. Nat. Methods.

[70] Kreshuk A, Zhang C (2019). Machine learning: Advanced image segmentation using ilastik. Methods Mol. Biol..

[71] Sommer, C., Strähle, C., Köthe, U. & Hamprecht, F. A. in *Eighth IEEE International Symposium on Biomedical Imaging *(*ISBI*), 230–233 (2011).

[72] Hiner MC, Rueden CT, Eliceiri KW (2017). ImageJ-MATLAB: A bidirectional framework for scientific image analysis interoperability. Bioinformatics.

[73] R: A Language and Environment for Statistical Computing (Vienna, 2020).

[74] Baddeley, A., Rubak, E. & Turner, R. *Spatial point patterns: Methodology and applications with R*.

