# Supplementary figures and images for: Cellular correlates of gray matter volume changes in magnetic resonance morphometry identified by two-photon microscopy
Source: Sci Rep. 2021 Feb 19;11:4234. doi: 10.1038/s41598-021-83491-8 (PMC7895945; doi:10.1038/s41598-021-83491-8)

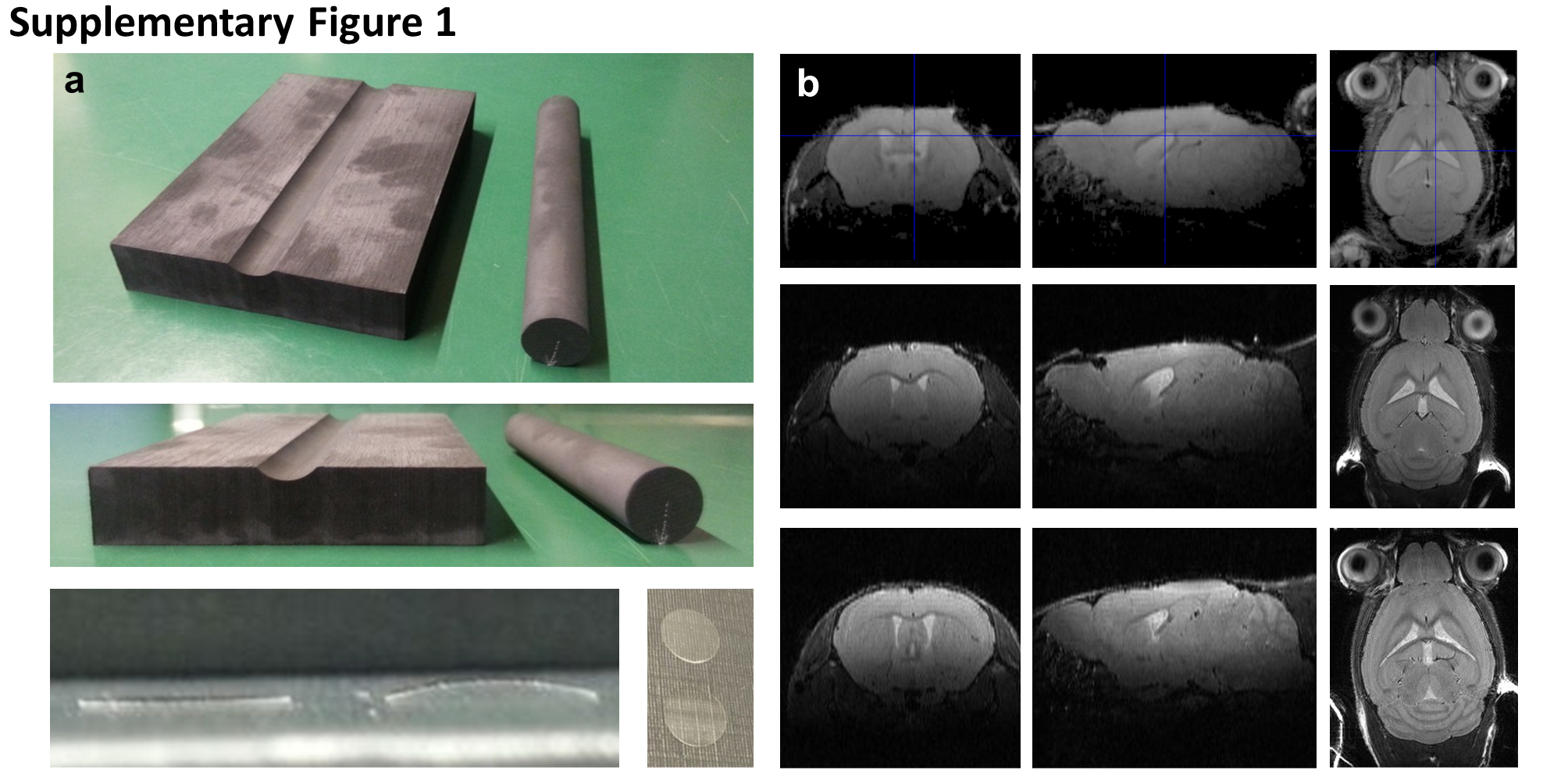

Supplement: Supplementary file 2 — Supplementary Figure 1. [file 41598_2021_83491_MOESM2_ESM.tif]

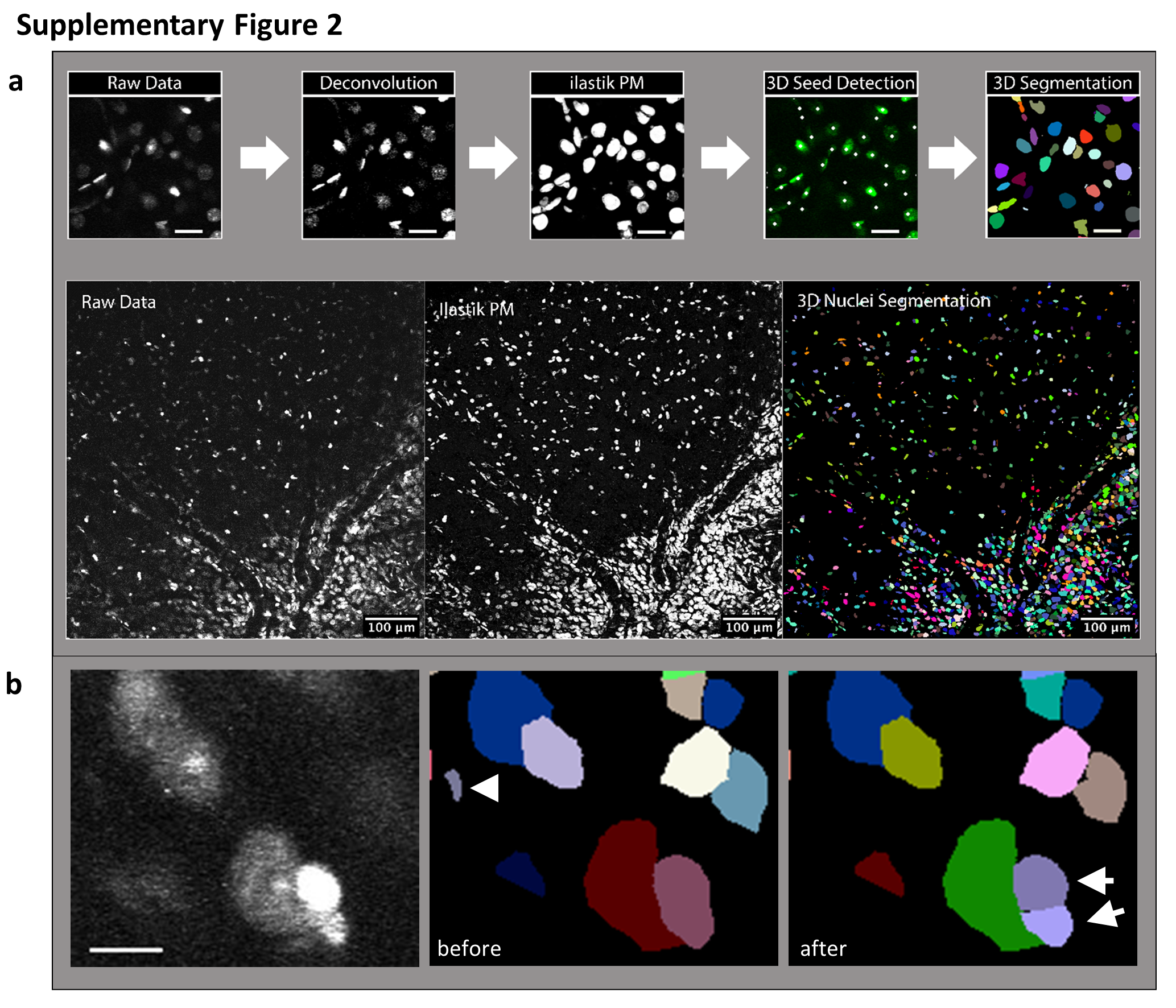

Supplement: Supplementary file 3 — Supplementary Figure 2. [file 41598_2021_83491_MOESM3_ESM.tif]

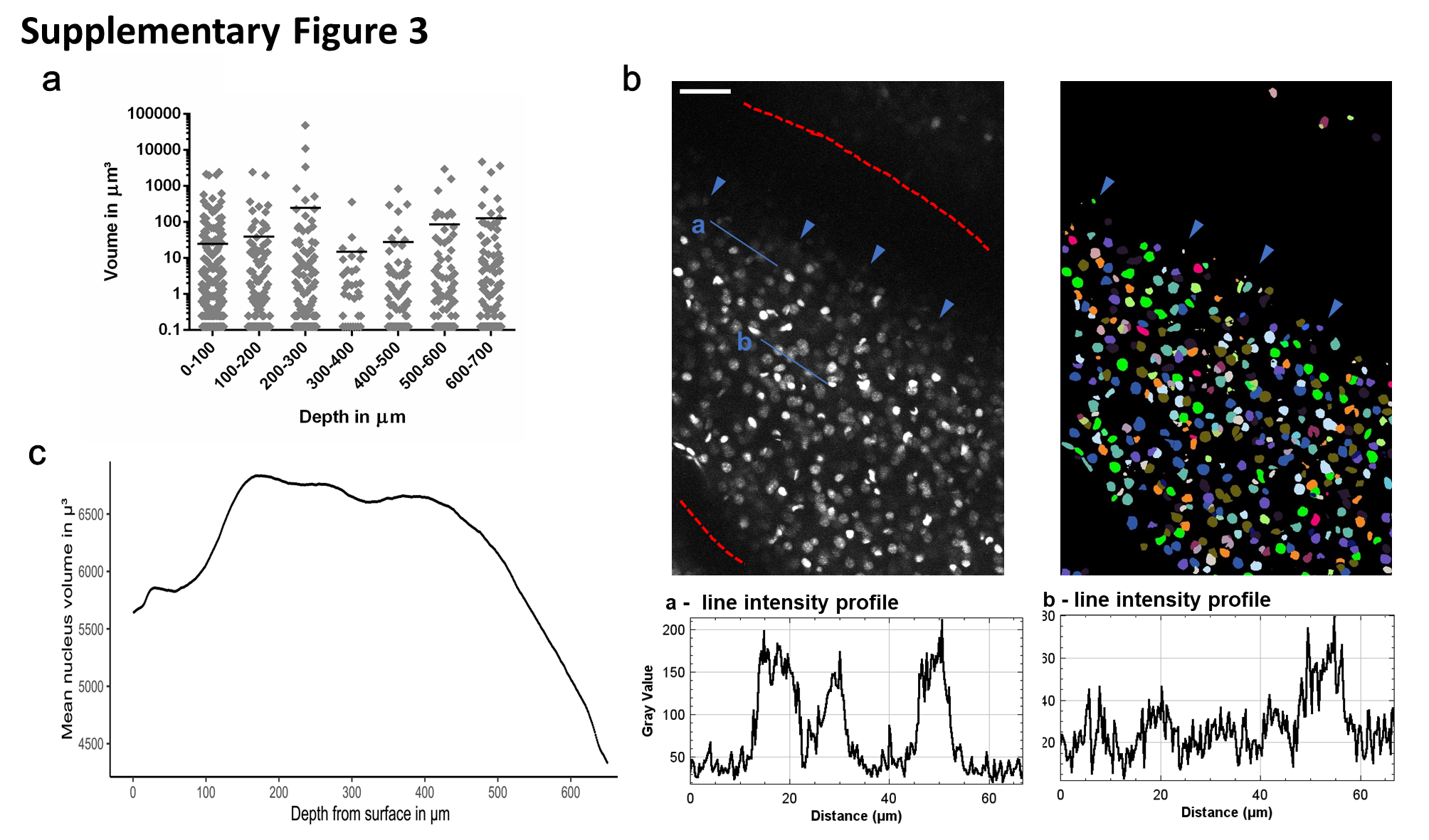

Supplement: Supplementary file 4 — Supplementary Figure 3. [file 41598_2021_83491_MOESM4_ESM.tif]

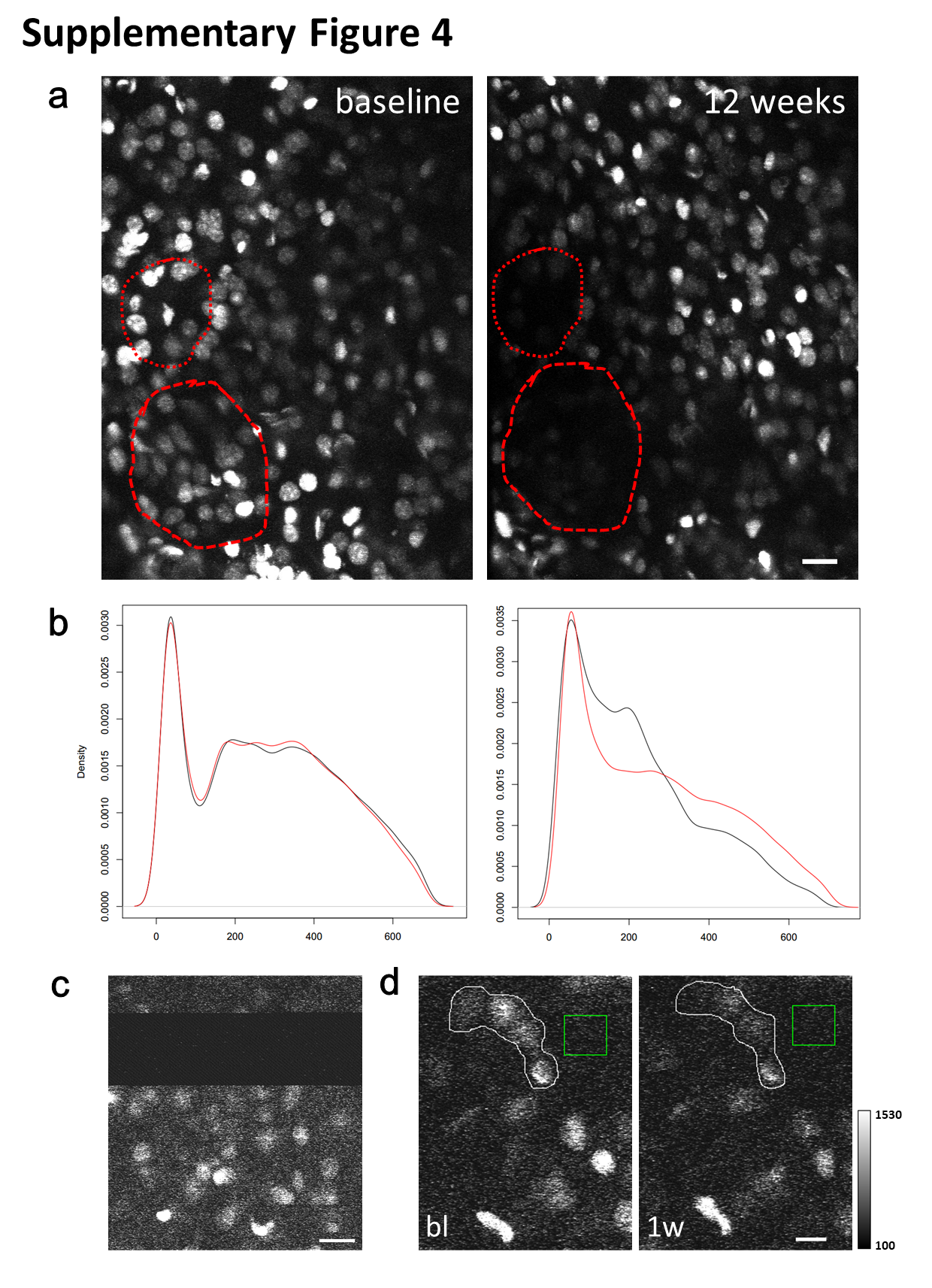

Supplement: Supplementary file 5 — Supplementary Figure 4. [file 41598_2021_83491_MOESM5_ESM.tif]

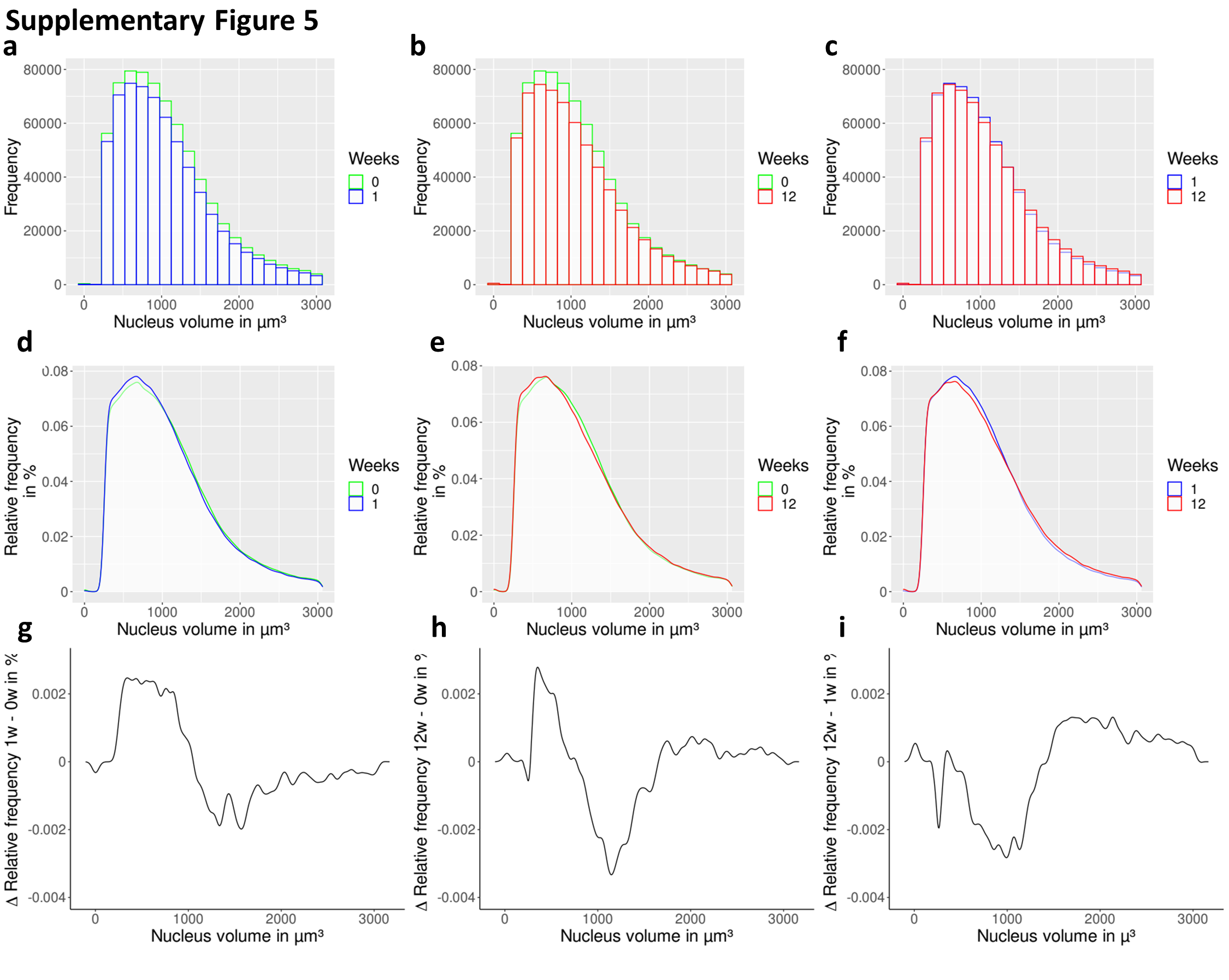

Supplement: Supplementary file 6 — Supplementary Figure 5. [file 41598_2021_83491_MOESM6_ESM.tif]

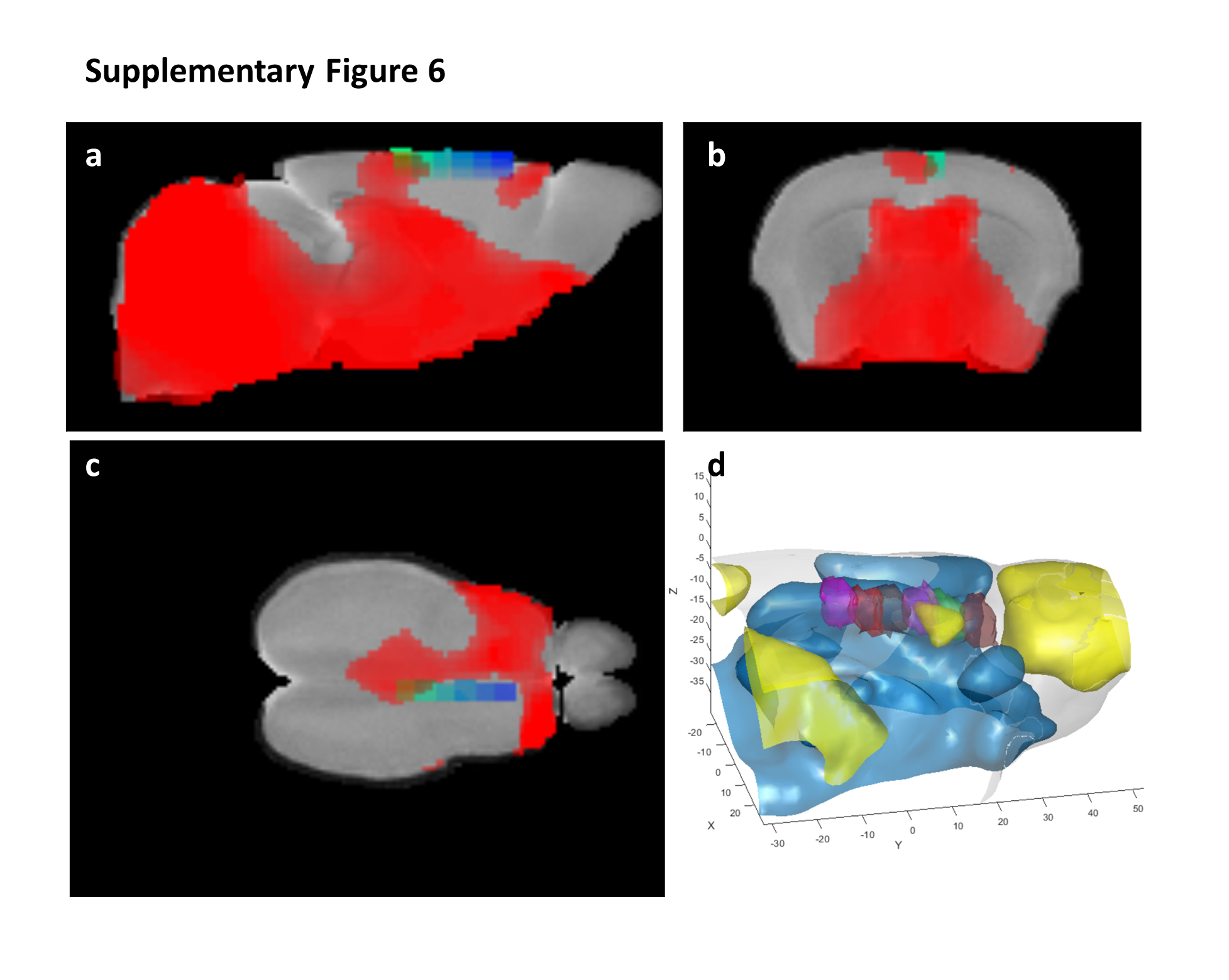

Supplement: Supplementary file 7 — Supplementary Figure 6. [file 41598_2021_83491_MOESM7_ESM.tif]
