# Supplementary material for: Cellular correlates of gray matter volume changes in magnetic resonance morphometry identified by two-photon microscopy
Source: Sci Rep. 2021 Feb 19;11:4234. doi: 10.1038/s41598-021-83491-8 (PMC7895945; doi:10.1038/s41598-021-83491-8)
Supplement: Supplementary file 8 — Supplementary Information. [file 41598_2021_83491_MOESM8_ESM.docx]

**Supplementary Material**

*for*

Cellular correlates of gray matter volume changes in magnetic resonance morphometry identified by two-photon microscopy

Livia Asan^1^, Claudia Falfán-Melgoza^2^, Carlo A. Beretta^1,3^, Markus Sack², Lei Zheng^2^, Wolfgang Weber-Fahr^2^*, Thomas Kuner^1^* and Johannes Knabbe^1,4^*

(1) Department of Functional Neuroanatomy, Institute for Anatomy and Cell Biology, Heidelberg University, Im Neuenheimer Feld 307, 69120 Heidelberg, Germany.

(2) Translational Imaging Research Group, Central Institute of Mental Health, Medical Faculty of Mannheim, University of Heidelberg, J5, 68159 Mannheim, Germany..

(3) CellNetworks Math-Clinic, Heidelberg University, Bioquant BQ001, Im Neuenheimer Feld 267, 69120 Heidelberg, Germany.

(4) Current address: Center for Psychosocial Medicine, Department of General Psychiatry, Heidelberg University Hospital, Voßstraße 4, 69120 Heidelberg, Germany.

* Corresponding Authors

Authors’ Emails and ORCIDs:

| L.A. | livia.asan@uni-heidelberg.de | https://orcid.org/0000-0001-5813-3242 |
| --- | --- | --- |
| C.F.-M. | claudia.falfan-melgoza@zi-mannheim.de | https://orcid.org/0000-0003-1313-6436 |
| W.W.-F. | wolfgang.weber-fahr@zi-mannheim.de | https://orcid.org/0000-0003-3503-7041 |
| C.A.B. | carlo.beretta@uni-heidelberg.de | https://orcid.org/0000-0002-6027-0796 |
| M.S. | markus.sack@zi-mannheim.de | https://orcid.org/0000-0002-3625-0672 |
| L.Z. | lei.zheng@uni-heidelberg.de |  |
| W.W.-F. | wolfgang.weber-fahr@zi-mannheim.de | https://orcid.org/0000-0003-3503-7041 |
| T.K. | thomas.kuner@uni-heidelberg.de | https://orcid.org/0000-0003-1896-9031 |
| J.K. | johannes.knabbe@uni-heidelberg.de | https://orcid.org/0000-0003-3170-111X |

|  |  |  |
| --- | --- | --- |

**Content:**

Legends of Supplementary Figures 1-6

Legend to Supplementary Video

Supplementary Methods

References for Supplementary Methods

**Supplementary Figure 1. Glass coverslips for cranial window were curved to match the natural brain curvature.**

**a** Bending of coverslips. A hollow was milled into a graphite block to match a 20 mm diameter graphite cylinder. Flat coverslips were lined up in the hollow, cylinder was placed on top and construct was heated in a laboratory oven.

**b** Comparing MRI scans of mice with a flat coverslip (top row), a bent coverslip (middle row) and a mouse without cranial window (bottom row). Less bulging of the brain at the craniectomy and a more natural curvature is achieved by the bent coverslip.

**Supplementary Figure 2. Analysis workflow for 3D nucleus detection and segmentation.**

**a** Automated image analysis pipeline and example single-plane images. Scale in upper panels= 20µm, lower panels= 100µm.

**b** Illustration of nucleus segmentation before and after the application of size filters. The minimum size filter removes small objects which most likely do not represent Histone-GFP positive nuclei, but autofluorescent lipofuscin granulae (white arrowhead). Objects with a volume over the maximum threshold are most likely wrongly merged and undergo re-segmentation, resulting in a correct split into two separate nuclei (white arrowheads). Maximum intensity projections of 16µm at a depth of 500µm. Scale = 10µm.

**Supplementary Figure 3. Factors influencing measured nucleus volume.**

**a** Mean of object volumes does not decrease with depth in a sample of fluorescently labeled bead clumps, indicating that the microscopy does not distort imaged volumes.

Beads were of 0,047um diameter (Invitrogen, catalogue #F8795, Excitation/Emission maxima 505/515nm) and distributed in a gel of 0,5% agarose mixed in PBS. In the resulting image, single beads and clots of beads show to be distributed throughout the sample. Images were recorded a voxel size of 0.25x0.25x2um with two-photon excitation at 960nm with the same objective lens as used in the *in vivo* study. Using Fiji, images were thresholded (Otsu’s method). Automated 3D size measurement of all particles was executed and the mean of the object volumes was calculated for bins of 100 um depth.

**b** Example image of vessel shadows and compromised image quality in their vicinity affecting detection and volume of nuclei in the 3D segmentation. Maximum Intensity projections of 20µm at 300µm depth. Dashed red lines: Vessel paths. Blue arrows indicate where the nucleus signal is diminished due to the localization in the vicinity of a vessel. Blue lines show the site of intensity profiles shown in a and b.

**c** Mean nucleus size vs. depth from surface over all study animals. Average of a moving window with a 50µm z-size. 0-150 µm: leptomeningeal layer and mostly glial cells of Layer 1. 150-450 µm: larger neurons of Layer 2/3, 450-700: L5 begins; mean nucleus size drops due to increased light scattering in deep tissue. Note: this area can also be included in longitudinal comparisons, when overall imaging conditions stay stable. Nucleus size comparisons across layers within one dataset, in contrast, cannot be performed.

**Supplementary Figure 4. Depiction of data exclusions in 2Pii.**

**a** Example of air blobs in the immersion water and subsequent signal impairment, making the two respective timepoints incomparable. Red circled dashed lines delineate area with diminished signal due to air at the 1 week image. Scale = 20µm. Maximum Intensity Projection of 20µm at 200µm depth.

**b** Relative density distributions of nucleus detections in a stack that was included in analysis (sum of density differences <500) and a stack that was excluded (>500; subsequent inspection showed air blob shadow in image).

**d** Example of a laser blankout in one frame leading to loss of signal.

**c** Example of the determination of SNR in a stack that was excluded from statistical analysis due to incomparable image quality. White line encloses signal reference area, background standard deviation was measured within green circle. SNR at baseline: 5.09, SNR at 1w: 4.50; Discrepancy in SNR: 11,5%. Single image plane at 300µm depth.

**Supplementary Figure 5. Details of nucleus volume change over time.**

**a-c**  Histograms compare the count of nuclei binned by size between all timepoints. Bin width = 150 µm³.

**d-f** Relative frequency distributions for changing nucleus volumes.

**g-i** Difference between relative frequencies of nucleus volumes on all timepoints.

**Supplementary Figure 6. 2Pii masks overlaid onto MRI whole-brain VBM results.**

2PII masks normalized to template space overlaid with the statistical map for the GMV increase after 12 weeks (TFCE, non-parametric test, 5000 permutations, color intensity is depicting -log10(p) for p<0.05 FDR corrected)

**a-c** Sagittal (a), coronal (b) and axial (c) views.

**d** Zoomed-in view of the location of 2Pii masks on the MRI volume.

**Supplementary Video. Visualization of the automated workflow for nucleus segmentation.**

The 3D rendering was created using arivis Vision4D software version 3.0.1 (arivis AG (Munich, Germany, https://imaging.arivis.com/en/imaging-science/arivis-vision4d). Raw data is displayed in green and probability map in gray. The centroids were imported as xyz coordinates from the output of the automated segmentation and visualized using random colours.

**Supplementary Methods: Details of VBM analysis.**

Longitudinal analysis was carried out by analyzing time points in a pairwise fashion. One analysis was conducted for baseline vs 1 week data, another was done for the baseline vs. 12 weeks data. This applies to all points after step 2 in the following step by step list. The pre-processing method is similar to longitudinal human VBM studies^1^.

VBM procedure step by step:

1. Rescale images by 10 to make them more compatible with SPM which is written for human data. This step does not change the data, it modifies the *.mat file defining the space of the image. It facilitates a big part of the SPM-functionality.
2. Rigid body registration of all images to the Paxinos space template from Biedermann et al.^2^ using the ‘co-register->Estimate only’ function in SPM. This step modifies the *.mat file, defining the space of the image, without changing the data.
3. Pairwise longitudinal non-linear registration between baseline scan with week 1 and week 12 scans, respectively, using the longitudinal registration toolbox (by Ashburner and Ridgway^3^). This procedure creates several images:
   1. A mean image (mid-point average) of both datasets after said longitudinal registration.
   2. Deformation fields of the non-linear registration that can be used to transform other images from the space of either of the measurements to the space of the average image.
   3. Images of the Jacobian rate, which is the difference of both Jacobian determinants from measurement A and B to the average image divided by time (which we set to 1 for simplicity) Change these in Figure 2.
4. All average images were skull-stripped with an in-house software based on Pulse-Coupled Neural Networks and similar to Chou et al.^4^
5. Tissue segmentation using the prior knowledge images from 13 into GM, WM, CSF (in native space). For this the mixed Gaussian model SPM segmentation was used (SPM12 -> old segmentation^5^).
6. The Jacobian rate of difference images from step (1) were multiplied by the average GM image from step (2) to derive a GMV rate of change map (GMV-C) depicting the volume change between the two timepoints (JR Modulated GM Image, figure 2C, middle Column).
7. The segmented GM and WM images were all put into Dartel (Diffeomorphic Anatomical Registration Through Exponentiated Lie Algebra toolbox in SPM12) to create group specific GM and WM Templates in the Paxinos space. This procedure creates so-called flow fields that depict the nonlinear transformation from the original images into the template space (figure 2, second to last column). The resulting flow fields were applied to the GMV-C maps for each subject to transform them into the template space without further modulation (Dartel-Normalise).
8. The final normalized GMV-C maps in template space were smoothed with a Gaussian filter (4 mm resized -> 0.4 mm original) and analyzed with a T-Test as well as non-parametric TFCE (Threshold Free Cluster enhancement, (Gaser, http://dbm.neuro.uni-jena.de/tfce). With 5000 Permutations and FDR correction.

**References for Supplementary Methods**

1 Meda, S. A. *et al.* Heavy Drinking in College Students Is Associated with Accelerated Gray Matter Volumetric Decline over a 2 Year Period. *Front Behav Neurosci* **11**, 176, doi:10.3389/fnbeh.2017.00176 (2017).

2 Biedermann, S. *et al.* In vivo voxel based morphometry: detection of increased hippocampal volume and decreased glutamate levels in exercising mice. *Neuroimage* **61**, 1206-1212, doi:10.1016/j.neuroimage.2012.04.010 (2012).

3 Ashburner, J. & Ridgway, G. R. Symmetric diffeomorphic modeling of longitudinal structural MRI. *Front Neurosci* **6**, 197, doi:10.3389/fnins.2012.00197 (2012).

4 Chou, N., Wu, J., Bai Bingren, J., Qiu, A. & Chuang, K. H. Robust automatic rodent brain extraction using 3-D pulse-coupled neural networks (PCNN). *IEEE Trans Image Process* **20**, 2554-2564, doi:10.1109/TIP.2011.2126587 (2011).

5 Ashburner, J. & Friston, K. J. Unified segmentation. *Neuroimage* **26**, 839-851, doi:10.1016/j.neuroimage.2005.02.018 (2005).
